# A candidate gene based approach validates *Md-PG1* as the main responsible for a QTL impacting fruit texture in apple (*Malus x domestica* Borkh)

**DOI:** 10.1186/1471-2229-13-37

**Published:** 2013-03-04

**Authors:** Sara Longhi, Martha T Hamblin, Livio Trainotti, Cameron P Peace, Riccardo Velasco, Fabrizio Costa

**Affiliations:** 1 Research and Innovation Centre, Foundation Edmund Mach, Via Mach 1, 38010, San Michele all’Adige, TN, Italy; 2 Institute for Genomic Diversity, Cornell University, 130 Biotechnology Building, 14853-2703, Ithaca, NY, USA; 3 Dipartimento di Biologia, Università di Padova, Viale G. Colombo 3, 35121, Padova, Italy; 4 Horticulture and Landscape Architecture, Washington State University, PO Box 646414, 99164-6414, Pullman, WA, USA

## Abstract

**Background:**

Apple is a widely cultivated fruit crop for its quality properties and extended storability. Among the several quality factors, texture is the most important and appreciated, and within the apple variety panorama the cortex texture shows a broad range of variability. Anatomically these variations depend on degradation events occurring in both fruit primary cell wall and middle lamella. This physiological process is regulated by an enzymatic network generally encoded by large gene families, among which polygalacturonase is devoted to the depolymerization of pectin. In apple, *Md-PG1*, a key gene belonging to the *polygalacturonase* gene family, was mapped on chromosome 10 and co-localized within the statistical interval of a major hot spot QTL associated to several fruit texture sub-phenotypes.

**Results:**

In this work, a QTL corresponding to the position of *Md-PG1* was validated and new functional alleles associated to the fruit texture properties in 77 apple cultivars were discovered. 38 SNPs genotyped by gene full length resequencing and 2 SSR markers ad hoc targeted in the gene metacontig were employed. Out of this SNP set, eleven were used to define three significant haplotypes statistically associated to several texture components. The impact of *Md-PG1* in the fruit cell wall disassembly was further confirmed by the cortex structure electron microscope scanning in two apple varieties characterized by opposite texture performance, such as ‘Golden Delicious’ and ‘Granny Smith’.

**Conclusions:**

The results here presented step forward into the genetic dissection of fruit texture in apple. This new set of haplotypes, and microsatellite alleles, can represent a valuable toolbox for a more efficient parental selection as well as the identification of new apple accessions distinguished by superior fruit quality features.

## Background

Fruit quality is defined by four main principal factors; appearance, flavour, texture and nutritional properties [1]. Of these factors, texture is the major component and the most important, especially for fruit with a crispy flesh [2], and due to its influence on general fruit quality. Texture decay causes substantial fruit loss during shipping and storage which is caused by the degradation of the internal cellular compartment of the fruit, which consequently promotes the development of diseases typical of the postharvest storage and shelf-life [3]. Texture is recognised as a complex set of different sub-phenotypes, which can be divided into two main categories [2,4]. The first encompasses mechanical features, and is fundamentally related to the strength exerted by the chemical bonds of the cell wall/middle lamella upon application of external pressure. The second category is defined by acoustic signatures, and is related to the cell wall breaking phenomenon with the consequent release of internal pressure [4,5]. Texture change is a physiological event which occurs naturally throughout fruit development and ripening [6], and the magnitude of texture decay is extremely variable between different apple varieties [7]. The variability observed is the result of physiological mechanisms activated during the fruit maturation and ripening, in which a large number of enzymes are co-ordinately expressed to remodel the cell wall/middle lamella polysaccharide structure, and regulated, amongst other factors, by the effect of ethylene and transcription factors [8,9]. The remodelling process is associated with a decrease in cell-to-cell adhesion, resulting in the separation of cells along the middle lamella (mealy texture) rather than a primary cell wall breaking (crispy texture; [1,7]) when the fruit is consumed. Fruit softening and textural changes thus involve a coordinated modification of the primary cell wall and middle lamella polysaccharide structure, a process which initially takes place with a dissolution of the pectin polysaccharides of the middle lamella, followed by a disruption of the ordered structure of the primary cell wall [10,11]. In several fruits the most active enzymes responsible for the pectin modification are polygalacturonase (PG) and pectin methylesterase (PME), while those acting on the primary cell wall are xyloglucan endotransglycosylase (XET) and expansin (Exp). Among this inventory, polygalacturonase is the major enzyme involved in the solubilization of the pectin polysaccharides [12-14]. The degradation of the cell wall/middle lamella architecture is in practice considered the final result of the concerted activity of these enzymes, which are usually encoded by multigene families, confirming the complex genetic control of the fruit texture metabolism [15-19].

Because of the impact that such physiological changes have on the marketability of edible fruit, researchers have for many years attempted to unravel the genetic basis of this mechanism, with the final goal of elucidating the genes underlying this dynamic process and the development of molecular markers suitable for phenotype prediction [6,20,21]. Quantitatively-inherited traits can be studied using a QTL mapping approach, which is generally carried out on bi-parental crosses. In apple, several reports have already identified major genomic loci putatively involved in fruit firmness and softening control [22-26], with the largest texture QTL mapping survey described by Longhi et al. [27]. However, QTL mapping carried out using full-sib progenies presents important limitations due to the number of alleles that can be simultaneously analyzed as the approach samples only a small portion of the total allelic diversity within the cultivated apple germplasm pool. Moreover, linkage analysis requires the development of a segregating population, making this procedure laborious and time consuming. In addition, in this type of material the number of recombination events per chromosome is generally low, limiting genetic mapping resolution [16,28,29]. To overcome these limitations, the analysis of a wider genetic background is rapidly becoming the main strategy for the dissection of complex genetic architecture in plants, establishing genotype-phenotype association complementary to bi-parental linkage mapping [30-36].

The main purpose of this study was to validate a QTL identifying a new set of valuable alleles associated to apple fruit texture sub-phenotypes in 77 cultivars. The phenotype was measured using an extremely precise technique to improve association resolution [37], and the impact of this gene on fruit texture was investigated further by cortex cell wall electron microscope scanning of two apple cultivars displaying contrasting texture phenotypes. Finally, a novel set of haplotypes and microsatellite marker alleles specifically related to important texture components are presented as valuable markers suitable for marker assisted parent selection (MAPS) as well to assist traditional breeding towards the selection of novel apple accessions characterized by superior fruit quality properties.

## Methods

### Plant material

A panel of 77 apple varieties, including both modern and old apple cultivars (Table 1), was chosen from two germplasm collections available at the Research and Innovation Centre of the Edmund Mach Foundation and the Laimburg Research Centre for Agriculture and Forestry, both located in the North of Italy (Trentino Alto Adige region). All the apple cultivars were planted in triplicate on M9 rootstocks and maintained following standard technical management procedures. Apple fruits were collected at the commercial harvest stage defined by monitoring the change of standard pomological parameters, such as skin and seed colour, brix value (total sugar content), cortex firmness assessed on site, and starch conversion index. Fruit were picked at a starch index of 7, based on a 1 to 10 scale.

**Table 1:** List of apple cultivars

**n°**	**Cultivar**	**a**	**b**	**Type**	**Alleles**	**n°**	**Cultivar**	**a**	**b**	**Type**	**Alleles**
1	Ambrosia	x		E	298-298	40	La Flamboyante (Mairac)	x	x	E	292-298
2	Ananas Renette	x	x	O	289-292	41	Ligol	x	x	E	292-292
3	Ariane (Les Naturiannes)	x	x	E	289-292	42	Limoncini	x	x	O	289-298
4	Ariwa	x	x	E	289-289	43	Magrè	x	x	O	289-298
5	Baumans Renette		x	O	289-289	44	Maigold	x	x	E	292-298
6	Bellida	x	x	E	289-298	45	Milwa (Junami)	x	x	E	292-298
7	Berner Rosen		x	O	298-298	46	Minnewashta (Zestar)	x	x	E	289-292
8	Baujade	x	x	E	292-298	47	Napoleone	x	x	O	298-298
9	Braeburn	x	x	E	289-298	48	Nevson (Sonya)	x	x	E	292-298
10	Brina	x	x	E	289-298	49	Nicogreen (Greenstar)	x	x	E	292-292
11	Gala Baigent (Brookfield)	x	x	E	292-298	50	Nicoter (Kanzi)	x	x	E	289-298
12	Calamari	x		O	289-298	51	Permain Dorato	x	x	O	289-298
13	Calvilla	x	x	O	289-292	52	Pilot	x	x	E	289-289
14	Caudle (Cameo)	x	x	E	289-292	53	Pinova	x		E	289-292
15	CIVG198 (Modì)	x	x	E	292-292	54	Red Delicious Camspur (Red Chief)	x	x	E	289-298
16	Civni (Rubens)	x		E	292-292	55	Red Elstar	x	x	E	292-298
17	Coop 39 (Crimson Crisp)	x	x	E	289-289	56	Red Field	x	x	E	289-298
18	MC38 (Crimson Snow)	x	x	E	289-292	57	Rome Beauty	x		E	298-298
19	Cripps Pink (Pink Lady)	x	x	E	292-292	58	Rosmarina Bianca	x	x	O	298-298
20	Cripps Red (Sundowner)	x	x	E	292-292	59	Gala Tenroy (Royal Gala)	x	x	E	292-298
21	Croncels	x	x	O	292-298	60	Rubinola	x	x	E	292-298
22	Dalinette (Choupette)	x	x	E	292-298	61	Sansa	x		E	298-298
23	Dalla Rosa	x	x	O	289-298	62	Santana	x	x	E	292-292
24	Delblush (Tentation)	x	x	E	292-292	63	Saturn	x	x	E	292-298
25	Delcorf (Delbarestivale)	x	x	E	292-298	64	Scarlet	x	x	E	289-298
26	Delearly	x	x	E	292-298	65	Scifresh (Jazz)	x	x	E	289-292
27	Delfloki (Divine)	x		E	289-292	66	Shinano Gold	x	x	E	292-298
28	Delorina (Harmonie)	x		E	289-298	67	Gala Schnitzer (Schniga)	x	x	E	289-292
29	Early Gold	x	x	E	298-298	68	Scilate (Envy)	x	x	E	292-292
30	Florina	x	x	E	289-289	69	Summerfree	x	x	E	292-298
31	Fuji	x	x	E	289-289	70	Tiroler Spitzlederer	x		O	289-292
32	Galmac	x	x	E	292-298	71	Topaz	x		E	292-298
33	Gelber Edelapfel	x	x	O	298-298	72	Weisser Wintertaffet	x	x	O	289-298
34	Gewürzluiken	x		O	289-298	73	Tavola bianca		x	O	289-298
35	Gloster	x	x	E	298-298	74	San Lugano		x	O	289-289
36	Golden Delicious	x	x	E	292-298	75	Renetta Champagne		x	O	298-298
37	Golden Orange	x	x	E	289-298	76	Coop 38 (GoldRush)		x	E	289-292
38	Granny Smith	x	x	E	292-292	77	Gold Pink (Gold Chief)		x	E	292-298
39	Red Delicious (Hapke Delicious)	x		E	289-298						

Apple cultivars are listed by name and trade mark (between brackets). N° is the code used to identify varieties in Additional file 3 and Figure 4. The letters “a” and “b” show the varieties used for the phenotypic assessments performed in years 1 and 2, respectively. “Type” indicates whether the variety is considered as old (O) or elite (E, new). The “Alleles” column shows the allelic size of the microsatellite marker Md-PG1_SSR_10kd for each cultivar. Allele 1: 289, allele 2: 292 and allele 3: 298.

Total genomic DNA was isolated from young leaf tissue, using the Qiagen DNeasy Plant mini kit (Qiagen) following the manufacturer’s protocol. DNA quantity and quality was measured spectrophotometrically with a Nanodrop ND-8000® (Thermo Scientific, USA).

### Apple fruit texture assessment

Fruit samples were stored in a controlled temperature cellar at 2°C for two months after harvest to maximize the trait phenotypic variance, as reported in Costa et al. [7], and high resolution phenotyping was carried out for two years. In order to avoid any effect of low temperature, samples were maintained at 20°C prior to analysis. Fruit texture was phenotypically dissected assessing simultaneously both mechanical and acoustic fruit profiles using a TA-XT*plus* texture analyser coupled with an AED – acoustic envelop device (Stable Micro System Ltd., Godalming, UK). Sample preparation, instrument settings and parameter characterization are described in detail in Costa et al. [7]. The fruit texture assessment was performed in an isolated room, avoiding any possible external noise interference. Texture profiles were analyzed with an *ad hoc* compiled macro (Exponent v.4, Stable Microsystems), which allowed the distinction of the texture sub-phenotypes using three main categories; mechanical, acoustic and force direction. General texture variability, expressed by the 14 parameters (Yield Force, Max Force, Final Force, Mean Force, Young’s Module, Area, N. of Force Peaks, Force Linear Distance, Force Ratio, Force Difference, N. of Acoustic Peaks, Acoustic Linear Distance, Max Acoustic Pressure, Mean Acoustic Pressure [7]), was illustrated by multivariate techniques, including principal component analysis (Statistica software v7).

### *Md-PG1* gene cloning and molecular marker genotyping

In this study a region of approximately 6 kb containing the *Md-PG1* gene was investigated. In particular, 2395 bp (representing the full length gene) and two regions of 800 bp each, located approximately 1 kb up and downstream from the start and stop codon respectively, were cloned and sequenced. The cloning was performed in order to design specific primers able to distinguish *Md-PG1* (MDP0000326734) from its homoeologue *Md-PG5* (MDP0000159240). *Md-PG1* fragment amplification was performed for the 1 kb up and downstream regions in a final volume of 25 μl with 10 ng DNA, 10× buffer, 0.25 mM dNTPs, 0.1 μM reverse and forward primers and 0.625 U of Eppendorf ® Taq polymerase. Amplification was performed using the following conditions: 2 min at 94°C, 32 cycles of 30 sec at 94°C, 30 sec at 56°C, 1 min at 72°C and a final extension of 7 min at 72°C. The full length *Md-PG1* gene was amplified in a final volume of 50 μl, with 10 ng DNA, 10× Advantage 2 PCR Buffer, 0.2 mM dNTPs, 0.4 μM reverse and forward primers and 50× Clontech® Advantage 2 Polymerase Mix. Amplification was performed following the manufacturer’s recommendations.

Candidate gene SNP genotyping was performed by re-sequencing (Sanger technology) the regions described above from the 77 apple cultivars, using specific forward and reverse primers listed in Additional file 1. Sequences were assembled and analysed with Pregap4 software version 1.3 (Staden Package). For fine mapping the *Md-PG1* region, in addition to the SNPs genotyped by re-sequencing, two microsatellites located in the assembled gene meta-contig were also used. The first was located 3 kb upstream of the *Md-PG1* start codon and retrieved from Longhi et al. [27]. The second SSR marker, here named Md-PG1_SSR_10kd (kd; kilobases downstream) was positioned at 10 kb downstream the stop codon, and was identified *de novo* using the software Sputnik (http://espressosoftware.com/sputnik/index.html). PCR for SSR marker genotyping was performed as reported in Longhi et al. [27] (Additional file 1). Fragment sizes were called by GeneMapper® (Applied Biosystems, by Life Technologies).

### Md-PG1_SSR_10kd mapping and QTL co-localization

The novel microsatellite motif found in the *Md-PG1* meta-contig was mapped to the framework map of the ‘Fuji × Delearly’ population [27] by specific primer sequences designed with the software Primer3 (http://primer3.sourceforge.net/). The marker was integrated employing the software JoinMap 4 [38], using a LOD of 5.0 and a recombination frequency of 0.45. To investigate the co-location of this marker with QTL regions already associated with texture dissected sub-traits, a MQM computation was performed *de novo* using MapQTL 6 [39], selecting Md-PG1_SSR_10kd as a co-factor in order to reduce the residual variance. A LOD threshold value of 3.0, established after running 1000 permutations, was chosen to consider a QTL significant. The linkage group was visualized using MapChart 2.1 [40].

### Population structure

To correct the analysis for population structure, the molecular profiles of 17 SSR markers (Additional file 2) and 368 SNPs [27] (16 out of the initial number of 384 failed to hybridize) were combined and used. Each microsatellite marker was selected according to map position, amplification efficiency and allelic size information available at the HiDRAS website (http://www.hidras.unimi.it). The population structure of the 77 apple cultivars was computed using a principal component analysis (PCA computed by Statistica software v7), which is a faster alternative to the MCMC model-based strategy, especially with large marker sets [34,41,42]. To account for genetic relatedness among individuals, the same marker data set used for population structure (Q matrix, fix effect) was also employed to generate a kinship matrix (K matrix), considered as random factor in the Mixed Linear Model performed using TASSEL [43,44].

### Linkage disequilibrium and marker-trait association

The linkage disequilibrium level among markers (SNPs and SSRs) identified within the apple cultivar collection, was calculated and visualised using Haploview 4.2, a software package designed for linkage disequilibrium statistics and haplotype block inference from genotype data [45]. This software was used to illustrate the pair-wise r^2^ among the 40 markers identified for the *Md-PG1* gene. To illustrate the LD decay within the *Md-PG1* region here investigated (from 3 kb upstream to 10 kb downstream the gene start and stop codon, respectively) the marker pair-wise r^2^ values were plotted against their physical distance on the chromosome 10. To fit the data, a smoothed line, represented by the logarithmic trend, was also added. The distribution of 63190 pairs of unlinked markers (368 SNPs) was employed to compute the r^2^, and the 95^th^ percentile was used as critical point to consider true the linkage between syntenic marker loci.

Marker-phenotype association analysis was performed using markers with a MAF ≥ 0.05 (minor allele frequency higher than 5%), and employing both a fixed general linear model (GLM) and a mixed linear model (MLM) with random factors. Initially, the GLM algorithm, implemented in the software package PLINK release 1.07 ([46]; http://pngu.mgh.harvard.edu/~purcell/plink/), was used to find associations between the marker set and the first two principal components (PC1 and PC2), derived by the PCA computation performed on the texture parameters. Genome-wide adjusted empirical *P-*values were then computed and corrected running 1000 permutations. In a second step, the same phenotypic and genotypic data sets were used to find association by implementing the MLM model of TASSEL, where *P-*values were corrected for false positives using the False Discovery Rate approach (FDR ≤ 0.05), performed by the QVALUE package implemented in R [47]. A *P-*value ≤ 0.05 was considered as the criterion for trait-phenotype association. MLM corrected by FDR was further used to exploit specific association between markers and each single texture dissected sub-phenotypes.

Considering that the phenotype variability is more likely associated with SNPs assembled in haplotype configuration rather than singularly, an additional analysis was performed with haplotypes, inferred by FastPhase [48] using only the significant SNPs. Haplotype-phenotype association was computed by the GLM algorithm, and *P-*values were adjusted by running 1000 permutations.

### Scanning electron microscopy of the apple cortex structure

To depict the different anatomic structure between a mealy (‘Golden Delicious’) and a crispy (‘Granny Smith’) apple fruit, a cortex portion from both cultivars was isolated and observed using a scanning electron microscope (SEM). Apple flesh slices were prepared by pulling apart the cortex portions which were then fixed for 2 hours at 4°C with 5% formaldehyde in a 0.1 M phosphate buffer (Na_2_HPO_4_ and NaH_2_PO_4_ pH 7). Samples were successively washed over night with 0.1 M phosphate buffer at pH 7 at 4°C. Dehydration was performed by incubating the slices for 15-20 min in solution with an increasing concentration of ethanol, and an Emscope 750 (Emitech, Ashford, Kent) was used to identify the critical drying point. Samples were finally coated with a SC 500 gold sputter coater (Bio-Rad Micro-science division) and examined using a Cambridge Instruments Stereoscan 260 scanning electron microscope.

## Results and discussion

### Apple fruit texture phenotype dissection

The 77 apple cultivars were phenotypically assessed for fruit texture by using a TA-XT*plus*-AED instrument. The trait dissection was performed identifying fourteen parameters over the combined mechanical-acoustic profile, ten of which were derived from the mechanical profile and four from the acoustic signature. The fruit texture variability evaluated within the apple collection over two years of observation is illustrated by the PCA plot (Additional file 3). The first principal component (PC1) describing 74.14% and 70.95% of the entire phenotypic variability for the two years respectively, together with the second principal component (PC2), accounting for an additional 12.44% and 12.95%, discriminated the orientation of the mechanical parameters from the acoustic group, suggesting a possible different genetic control for these two components [7]. The variable projection on the PCA space distinguished the separation between the two general texture components (Additional file 3), with all the mechanical parameters plotted in the negative PC1 and positive PC2 graph area, and the acoustic more oriented towards the area characterized by negative value for both PCs. The consistent variable orientation and cultivar distribution between the two years confirms this novel strategy as an efficient and reliable method to dissect the fruit texture complexity. In both years, the data distribution clearly distinguished mealy varieties (such as ‘Delearly’, ‘Golden Delicious’, ‘Gelber Edelapfel’, plotted on the positive PC1 values) from known firm and crispy varieties (such as ‘COOP39’, ‘Granny Smith’, ‘Fuji’ and ‘Cripps Pink’) placed in the area characterized by negative PC1 values.

### Candidate gene based marker genotyping

The apple genome underwent a recent duplication resulting in a pair-wise colinearity of large chromosome segments [49] and because of this *Md-PG1*, the candidate gene investigated in this work and located on chromosome 10, shows a similarity of 86% with its homoeologue *Md-PG5* on chromosome 5 [27]. To enable the characterization of the specific sequence for *Md-PG1*, the sequences of the two genes were retrieved from the ‘Golden Delicious’ genome.

Out of the 38 SNPs genotyped over the *Md-PG1* genomic region, 22 were identified by re-sequencing the full length (2395 bp) within the apple collection, with an average frequency of 1 SNP/108.9 bases. Among them, ten were located in exons (total length of 1380 bp) with a frequency of 1 SNP/138 bp, and 12 SNPs in introns (total length of 1015 bp) with a frequency of 1 SNP/84.5 bp. These frequencies are consistent with previous observation made for apple of 1 SNP/149 bp [50], as well as in other outcrossing plant species such as pine with 1 SNP/102 [51], but lower than white clover (1/59, [52]) and grapevine (1/64, [53]). From the gene structure analysis resulted that SNPs found in non-coding regions were two fold more frequent than in coding ones [54]. Within the *Md-PG1* predicted gene (with an intron/exon structure consistent with Bird et al. and Atkinson and Gardner [55,56]) the SNP’s location along with their functional annotation is presented in Additional file 4 and Additional file 5. Among the remaining sixteen SNPs, three were located in the 3^′^ UTR region, two were found 1 kb upstream the start codon and eleven 1 kb downstream the stop codon of the gene. In addition to the 38 SNPs, two microsatellite markers were also included. The first (Md-PG1SSR) was retrieved from the data of Longhi et al. [27], while the second, named Md-PG1_SSR_10kd, was *de novo* identified screening for microsatellite repetition over the *Md-PG1* genomic contig (MDC000024.376 and MDC004966.443, available at http://genomics.research.iasma.it).

### Md-PG1_SSR_10kd co-localizes with a texture hot-spot QTL

**Figure 1:**
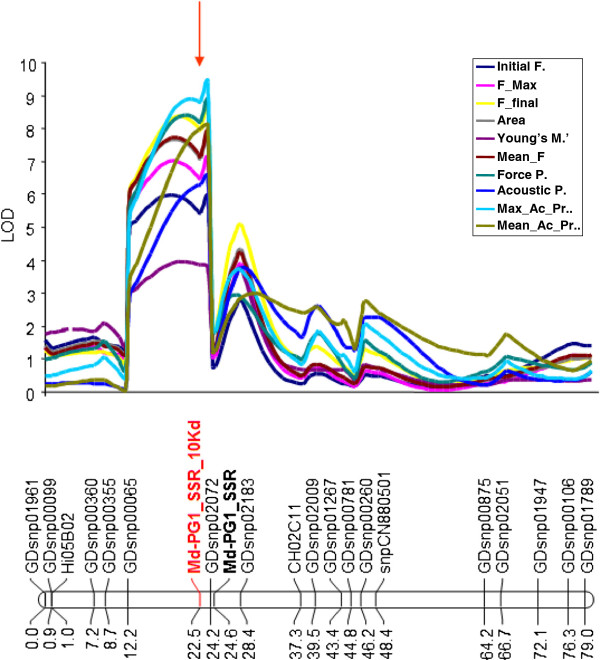
**QTL-LOD profile for fruit texture dissected traits on LG 10.** The different texture sub-traits are visualized with different colours, while the red arrow points to the position coincident with the Md-PG1_SSR_10kd locus. In the linkage group chart, the microsatellite marker used to map the *Md-PG1* gene and presented in Longhi et al. [27] is shown in bold black text, whereas the novel microsatellite marker named Md-PG1_SSR_10kd is presented in bold red text.

The newly developed Md-PG1_SSR_10kd microsatellite marker was further amplified and integrated into the ‘Fuji × Delearly’ genetic map, where several QTLs for apple fruit texture were previously mapped [27], and among which the major hot-spot cluster coincided with *Md-PG1* gene. The allele segregation allowed the mapping of this marker in the same position of the gene, at 22.5 cM from the top of the linkage group. This second version of the ‘Fuji x Delearly’ map was used to calculate an improved version of the QTL profile for fruit texture, implementing this new marker as co-factor for the multiple QTL detection (MQM algorithm). The QTL cluster was confirmed on chromosome 10 (Figure 1) and associated with ten sub-traits representative of the fruit texture, such as yield force, maximum, final and mean force area, Young’s module, number of force and acoustic peaks, mean and maximum acoustic pressure. It is interesting to note that the highest LOD value corresponded with this novel marker, with a LOD value ranging from 3.85 to 8.80 and expressing a phenotypic variance between 19% and 41.8%, thus confirming its impact in the fruit texture association. Among the texture parameters employed in the QTL mapping, the Young’s module, related to flesh elasticity, showed the lowest level of association. This is consistent with the observations of Costa et al. [7] about this feature, which reported this index as more related to the cell layer compression behavior rather than the cell wall fracturing, the causal event of the mealy/crispy fruit texture, thus under the control of other genes encoding cell wall degrading enzymes.

### QTL validation and allelic survey within an apple collection

**Figure 2:**
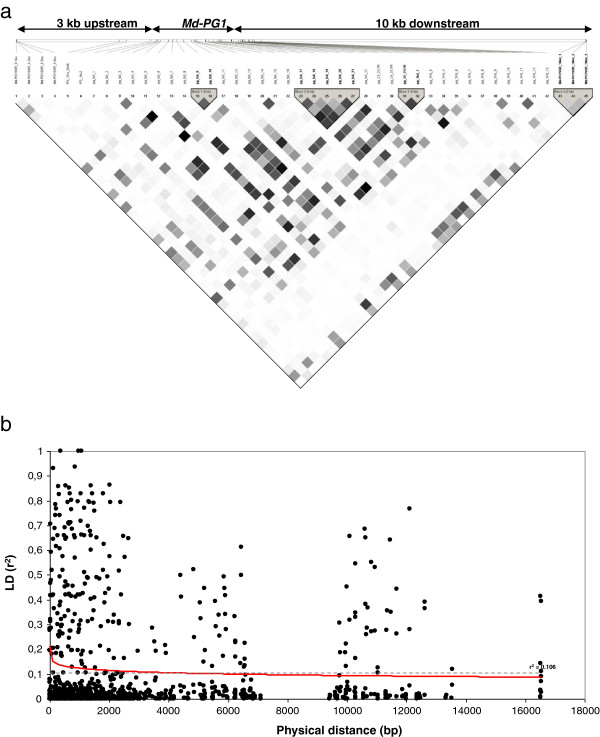
**LD plot with haploblock structure and LD decay within the*Md-PG1*region.** The LD image is based on r^2^ values (**a**), the darker the colour the higher the value. In the upper part, the number of polymorphic sites and the markers considered for the computation are indicated. The four haploblocks are highlighted in grey. On the top the physical position of the markers on the *Md-PG1* contig are also illustrated, on which are distinguished the three main portions: 3 kb upstream, gene full length and the 10 kb downstream. Markers in bold are those included in the four haploblocks. Panel “**b**” shows the scatterplot of the LD decay of the linked marker pairs on chromosome 10 as a function of the physical distance (bp). The red line indicates the data fitting line. LD threshold, based on the 95^th^ percentile of the unlinked marker pair distribution is superimposed on the scatterplot and represented by a grey dashed line.

A collection of 77 apple cultivars was analyzed using 38 SNPs genotyped by re-sequencing and two SSR markers by PCR amplification, contained in a region of approximately 16 kb. Among them, 22 SNPs were associated with the *Md-PG1* full length, while the other 16 were identified in two genomic portions of approximately 1 kb each, respectively located 1 kb up and downstream of the coding portion of the gene. Finally, the two SSR markers additionally delimited two outer regions, located 3 and 10 kb up and downstream of the gene. LD analysis, performed in order to estimate the non-random association among the 990 pairs of polymorphic sites over the *Md-PG1* region, was computed by HaploView (Figure 2a). Four haploblocks were identified, defined by markers showing a r^2^ between 0.19 and 0.85. The first block included two SNPs positioned in two adjacent exons, the second comprised SNPs in both exons and introns, while the other two blocks contained markers identified after the *Md-PG1* stop codon. The third haploblock contained two SNPs, one located in the 3^′^ UTR region and 1 kb downstream, respectively. The fourth block was represented by the Md-PG1_SSR_10kd microsatellite, located at 10 kb downstream the *Md-PG1* stop codon. The LD decay over the *Md-PG1* region (Figure 2b) was plotted in reference to the LD base-line set at r^2^ = 0.106, represented by the 95^th^ percentile of the r^2^ distribution of unlinked markers. The baseline was determined following the methods of Breseghello and Sorells [57], which proposed that LD extent should be defined comparing the target LD with the one observed among unlinked loci, being the LD dependent on the sampling scheme. In this computation the intersection between the data fitting curve and the LD baseline defined an LD extent of ~2 kb, pointing to a rapid LD decay within this gene, confirming the suitability of the candidate gene approach [58] to find association between fruit texture and markers based on *Md-PG1*. Among the set of 40 markers only those having a MAF value higher than 5% were further used to find association with the texture phenotype, and to avoid spurious associations (due to false positive effects), the structure was taken into consideration as covariant. When phenotypic traits are correlated with population structure, loci that are not related to the trait under investigation may nonetheless be statistically associated [33]. Statistical correction for multiple test and MAF ≥ 0.05 were also employed in order to improve the QTL detection confidence with small sample set. For a better estimation of the size effect of this QTL, a wider collection will be further assembled and implemented in the analysis.

The genetic relationship among the 77 apple cultivars was investigated by Principal Component Analysis. From the total number of PCs, ten were finally selected as covariates to represent the population structure, accounting for 32% of the total genetic variance. The traits employed in the association were represented by a phenotypic dataset containing 14 texture parameters, clustered in two main categories, mechanical and acoustic. These two groups, distinguished by the two principal components (computed on the phenotypic data set), captured 85% of the total phenotypic variance. Initially, the analysis considered the first two PCs as traits, and the association with markers (MAF ≥ 0.05) was computed by running both GLM and MLM modules. Six markers were commonly identified by both algorithms as statistically associated with PC1, consistent with the higher textural variation explained by this component compared to PC2 (Table 2). Among this set, five SNPs are specifically located within the full length *Md-PG1* gene, and three are included in haploblocks. In particular, PG-full 10 is located in the 1^st^ haploblock, and PG-full 19 and 20 in the 2^nd^ haploblock. The remaining two SNPs, PG-full 1 and 12, were not present in any of the haploblocks defined here. This association additionally confirmed the role of one SNP in particular, here named PG-full 1. This SNP was in fact originally used to map *Md-PG1* to linkage group 10 [26,27]. The effect of this marker is here validated in a wider germplasm collection, supporting the previously formulated hypothesis about the effect of the amino acid changed due to this SNP on the fruit firmness control [26]. It is worth noting that the last marker of this set is the microsatellite Md-PG1_SSR_10kd, located in the 4^th^ haploblock. In the MLM computation (corrected for false discovery) this microsatellite was also associated with PC2 (q-value: 0.035739, not shown in the table), principal factor expressing a lower quotient of phenotypic variability, but oriented towards the phenotypic dissection of the mechanical/acoustic components.

**Table 2:** Markers statistically associated with the mean PC1 computed for the two years of phenotypic observations

***Marker***	***EMP2***	***qvalue***
pg_full_1	0.01998	0.012097
pg_full_10	0.04096	0.010826
pg_full_12	0.02398	0.011884
pg_full_19	0.04895	0.015247
pg_full_21	0.04096	0.010826
Md-PG1_SSR_10kd-3	0.008991	0.006551

GLM associations are indicated by EMP2 (genome-wide empirical value, corrected by 1000 permutations), while MLM associations are indicated by the q-value (*P-*value corrected by FDR < 0.05).

To better exploit the association between the markers and fruit texture sub-traits, each SNP was further analyzed with each single texture parameter. Eight out of the fourteen texture components resulted statistically associated with the marker set employed in the analysis (Additional file 6), including acoustic linear distance, number of force and acoustic peaks, area, final force, yield, maximum and mean force. The remaining six parameters showed a limited number of associated markers. Maximum and mean acoustic pressure were associated only with PG-full 9 and 1 kb down 5. Force linear distance was associated with PG-full 9 and 1 kb down 5, while Young’s module showed a significant *P-*value only with the third allele of Md-PG1_SSR_10kd. Parameters related to the force direction (∆ force and force ratio) were associated with PG-full 1, 12, 13 and the allele 2 and 3 of the Md-PG1_SSR_10kd marker.

**Figure 3:**
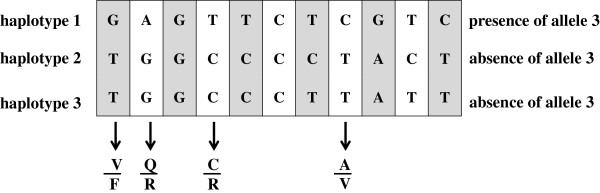
**Structure of the three Md-PG1 haplotypes.** For each haplotype the respective association with the presence or absence of the allele “3” of the microsatellite marker Md-PG1_SSR_10kd is reported. At the bottom, the four significant amino acid changes, differentiating haplotypes 2 and 3 from haplotype 1, are highlighted.

To estimate more accurately the SNP frequency markers assembled into haplotypes were tested for association with the texture sub-traits. From the total number of SNPs significantly associated to the texture components, eleven, with a MAF ≥ 0.05 and located in the *Md-PG1* full length, were selected and used to infer three significant haplotypes (H1, H2 and H3; Table 3 and Figure 3). H1 (the most frequent) showed a relevant association with nine texture sub-traits, and it was shared by cultivars distributed in the PCA plot over the PC1 axis, thus characterized by medium/low texture behaviour (mealiness, like ‘Golden Delicious’). H2, associated to six texture sub-traits of both a mechanical and acoustic nature, characterized cultivars known for a favourable texture properties (crispness), such as ‘Cripps Pink’, ‘Granny Smith’ and ‘Nicogreen’. The last haplotype H3, was associated with only two texture sub-traits (10.7% of the explained phenotypic variance), but it is worth noting that these are specifically related to the acoustic components (acoustic linear distance and number of acoustic peaks). As with H2, H3 was present in high texture performing apple cultivars, such as ‘CIVG198’, ‘Coop39’, ‘Ligol’ and ‘Minnewashta’. H2 and H3 also share four SNPs which leads to changes in the *Md-PG1* primary sequence. These changes have been analyzed in order to see whether they might have an impact on the polygalacturonase enzyme activity, explaining, at least partially, the high flesh firmness typical of the varieties harbouring these two haplotypes. SNP1 (V/F) is located in an un-conserved region and F is one of the most frequent residues, thus it is not expected to negatively influence PG activity (Additional file 7). On the contrary, both the Q/R (SNP6) and the C/R (SNP10) conversions might slightly change the *Md-PG1* activity. Indeed R residues are very rare among plant PGs in both positions. The last considered substitution (A/V, SNP18) is closed to a highly conserved region, with the A as the predominant amino acid, while V being slightly bigger and more hydrophobic might decrease the PG activity. As the alleles leading to the three changes are homozygous in all the tested crispy varieties (exception made for ‘Minnewastha’, which is heterozygous for the only SNP6), we hypothesize that a less active polygalacturonase isoenzyme could be less effective in middle lamella depolymerization. This finding was moreover supported by the fact that apple cultivars having a homozygous presence of the haplotype H1 are characterized by an extremely low texture property (mealiness), such as ‘Dalla Rosa’, ‘Early Gold’, ‘Limoncini’, ‘Napoleone’, ‘Permain Dorato’, ‘Rosmarina Bianca’ and ‘Tavola Bianca’ (a set represented for the most by old apple varieties).

**Table 3:** Association between the three significant haplotypes and texture sub-traits

		***Acoustic linear distance***	***Acoustic peaks***	***Area***	***FinalForce***	***Force Peaks***	***MaxForce***	***Mean Force***	***Yield Force***	**Young Module**
***Haplotype***	***%***	***EMP2***	***EMP2***	***EMP2***	***EMP2***	***EMP2***	***EMP2***	***EMP2***	***EMP2***	**EMP2**
H1	2.4-20.3	**0.00100**	**0.00300**	**0.00100**	**0.00100**	**0.02997**	**0.00200**	**0.00100**	**0.00300**	**0.03996**
H2	0.7-8.3	**0.01698**	**0.03696**	**0.02697**	0.08591	0.08991	**0.04895**	**0.02198**	**0.03796**	0.07093
H3	0.7-10.7	**0.01898**	**0.01598**	0.66330	0.47250	0.35760	0.60040	0.62540	0.79120	0.98900

In bold significant association values with a *P*-value < 0.05 after correction of 1000 permutations are reported.

### Parental selection

**Figure 4:**
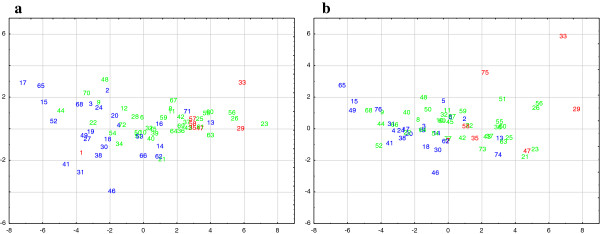
**PCA distribution in correlation with Md-PG1**_**SSR**_**10kd.** Numbers correspond to the varieties as reported in Table 1. Colours indicate the allelic dosage for Md-PG1_SSR_10kd-3, with blue used for cultivars characterized by the absence of the “3” allele, green for cultivars having this allele in heterozygous state and red for cultivars carrying this allele in a homozygous state (thus present two times).

These SNPs and haplotypes can be considered as a novel toolbox to improve the phenotype prediction efficiency of breeding programs towards the programmed identification of the most suitable parents (MAPS-marker assisted parent selection) and the subsequent selection of novel accessions (MASS-marker assisted seedling selection) with improved fruit texture quality. It is also worth emphasizing, as markers useful for breeding, the microsatellite Md-PG1_SSR_10kd which was highly associated to the set of texture sub-traits. This microsatellite was targeted in the *Md-PG1* meta-contig*,* in strong LD with SNPs located within the gene. The allelic state configuration of this marker within the apple cultivars (Table 1) showed a clear dosage effect when compared to the texture distribution over the PCA plot for both years (Figure 4 and Additional file 8). Apple cultivars characterized by the homozygosity for the allele Md-PG1_SSR_10kd_3 were located in the positive PC1 area of the PCA, thus showing a general low texture behaviour. When this allele was absent the cultivars, distinguished by PC1 values from -8 to 0, showed a superior textural properties. In contrast, apple cultivars characterized by a heterozygous state for allele “3” showed an intermediate texture distribution. As additional proof of the utility of this microsatellite marker for texture selection programs in apple, a correlation with the three significant haplotypes was also observed. Apple cultivars characterized by H2 and H3 (the two favourable haplotypes associated with a valuable texture performance) lack the allele “3” of this microsatellite marker, which showed a dosage effect associated to fruit texture decay. The haplotype survey carried out on the 77 apple cultivars, and the validation of their association with the texture components, highlighted that cultivars showing the two haplotypes H2 and H3, or lacking the allele “3” of the microsatellite marker, are distinguished by a favourable fruit texture behaviour. These varieties (Table 1) are already employed as valuable potential parents in breeding programs addressing the improvement of fruit quality in apple, while the SSR alleles/haplotypes can be further exploited to investigate the breeding potential of other apple accessions not yet characterized.

### Apple fruit cortex structural characterization

**Figure 5:**
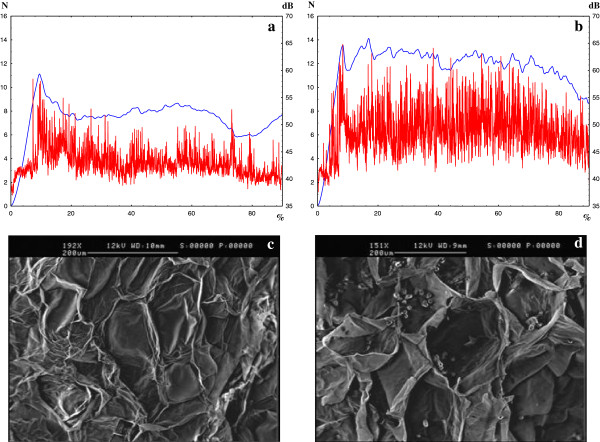
**Texture profiling and cortex scanning in ‘Golden Delicious’ and ‘Granny Smith’.** Panel **a** and **b** illustrate the combined mechanical (blue line) and acoustic (red line) profile for ‘Golden Delicious’ and ‘Granny Smith’, respectively. Scanning electron microscopy of the fruit cortex cells, sampled after one week of postharvest shelf-life ripening, is shown for ‘Golden Delicious’ (**c**) and ‘Granny Smith’ (**d**). The scale bar is reported on the top of both panels.

The impact of the *Md-PG1* gene on fruit development and ripening was also investigated by SEM (scanning electron microscopy). The mealy/crispy texture behaviour of the two cultivars was assessed using the texture analyser (Figure 5a and b). The analysis of the combined texture profiles (mechanical and acoustic), performed at a ripe stage, showed that ‘Golden Delicious’ displayed a lower texture performance (mealiness) with respect to ‘Granny Smith’, in which a better texture behaviour was observed (crispness). The digital extraction of the parameters underlined the different textures of these two cultivars showing a maximum force of 11.12 and 14.11 N, and acoustic peaks of 13 and 104 for ‘Golden Delicious’ and ‘Granny Smith’, respectively.

The polysaccharide depolymerization of the middle lamella is one of the major events distinguishing mealy from crispy cultivars, and excessive degradation controlled by the polygalacturonase enzyme determines a significant weakening of the chemical binding between adjacent cells, facilitating cell-to-cell slippage along the middle lamella upon mechanical compression. This hypothesis is consistent with the fruit cortex structural observation made by SEM. Fruit cortex cells of ‘Golden Delicious’ had generally collapsed, due to a loss of internal turgor pressure, but were structurally intact, meaning mechanical rupture followed the cell boundaries at the level of the middle lamella, which in this cultivar were highly degraded. In ‘Granny Smith’, the cells were completely broken, showing an increase in laceration of the cell walls rather than in the middle lamella, most likely due to a reduced degradation activity that prevented cell separation occurring (Figure 5c and d).

The distinct anatomical structure between the two cultivars are also correlated with the different haplotype structure found within the *Md-PG1* gene. It is worth noting that both cultivars present the Md*-*PG1 haplotypes in heterozygous state. One is a common haplotype shared between the two, which is not statistically associated to any fruit texture parameters. The other haplotype is represented by H1 and H2 for ‘Golden Delicious’ and ‘Granny Smith’, respectively, both distinguishing particular fruit texture behaviours.

## Conclusion

The results of this work validated the impact of a QTL associated to fruit texture in apple presenting a new set of *Md-PG1* alleles valuable for a marker assisted parent selection. Fruit texture is one of the principal quality factors in apple, and is a priority world-wide in modern apple breeding programs. Many works have been already presented to the scientific community, generally limited to QTL surveys focused on bi-parental maps. In this study, we identified a new set of markers and haplotypes related to *Md-PG1* gene and associated to texture dissected sub-traits. In particular three haplotypes and a novel microsatellite marker, with a clear allelic dosage effect, were specifically associated to several texture components. The fruit texture dissection in mechanical and acoustic sub-phenotypes allowed more detailed and precise phenotyping to be performed in this investigation, which to date has been recognised as the limiting step in association studies. Among the genes devoted to cell wall metabolism, *Md-PG1* is one of the most relevant genes encoding a cell wall degrading enzyme, and the results discussed here represent a first step towards the genetic dissection of the mechanical-acoustic component of fruit texture, a fundamental aspect for apple breeders, since the acoustic response (crispness) is generally recognised as the main priority in the creation of new elite accessions.

## Competing interest

The authors declare that they have no competing interests.

## Authors’ contributions

SL performed the molecular work and drafted the manuscript; MTH supported the association analysis and edited the manuscript; LT contributed to SEM and PG1 sequence analysis; CPP participated to the genotyping; RV provided access to the apple genome; FC designed the experiment, supervised the work and finished the manuscript. All authors read and approved the final manuscript.

## Supplementary Material

Additional file 1 **List of primers used for gene cloning and sequencing as well as sequences of the two microsatellite markers identified within the*Md-PG1*region, and located at 3 kb upstream (Md-PG1SSR) and 10 kb downstream (Md-PG1**_**SSR**_**10kd) from the start and stop codon, respectively.** Click here for file

Additional file 2 **List of 17 SSRs used to compute the population structure.** For each marker the forward and reverse primer sequences and linkage group are provided. For the list of SNPs see Longhi et al. [27]. Click here for file

Additional file 3 **PCA plot (first two dimensions) distribution of the varieties assessed with the novel texture phenomics strategy at postharvest stage for year 1 (A) and 2 (C) respectively.** On the variables projection (panel B and D) “a” and “f” means acoustic and force parameters respectively, while “d” is for force direction. Panels E and F show the eigenvalue for the two years of assessment. Numbers: variety codes as reported in Table 1. Click here for file

Additional file 4 **SNP organization within*Md-PG1*gene.** For each SNP used in the association analysis the location with respect to the gene structure (location), the type of aminoacid substitution (type; nc: non-coding, ns: non-synonymous, s: synonymous) and the change of aminoacid and physical position (in bp after the start codon ATG) are reported. Click here for file

Additional file 5 **Md-PG1 amino acid sequence derived by the predicted Md-PG1 sequence.** The synonymous substitutions are shown in blue, while the non-synonymous substitutions are shown in yellow. (PPT 179 kb) Click here for file

Additional file 6 **Significant marker – texture sub-traits association computed with MLM algorithm and corrected by FDR < 0.05.** Only significant values (*P*-value < 0.05) are reported. Ac is for Acoustic parameter, while F is for Force-mechanical parameters. Click here for file

Additional file 7 **Amino acid sequence alignment of the different PG enzymes derived from Md-PG1 (MDP0000326734) alleles with other plant PGs.** The consensus Md-PG1 sequence is here indicated as PG1p-gen and is aligned with the amino acid sequence of the three haplotypes H1, H2 and H3 (here indicated as PG1p_h1, PG1p_h2 and PG1p_h3, respectively) and the Md-PG1 sequence already present in public databases (Acc. no.: P48978). Other sequences are indicated with the species initials followed by the Acc. nos (e.g.: Pc_BAC22688.1 stands for *Pyrus communis* BAC22688.1). Apple, (*Malus xdomestica*, MDP), peach (*Prunus persica*, ppa) and Arabidopsis (*Arabidopsis thaliana*, AT) sequences have been downloaded from Phytozome (http://www.phytozome.net), while the others have been downloaded from NCBI (http://www.ncbi.nlm.nih.gov/entrez). Species used in the alignment are: *Malus xdomestica* (Md), *Pyrus communis* (Pc), *P. pyrifolia* (Ppy), *Actinidia chinensis* (Ac), *A. deliciosa* (Ad), *Prunus persica* (Pp), *Ricinus communis* (Rc), *Diospyros kaki* (Dk), *Vitis vinifera* (Vv), *Populus trichocarpa* (Pt), *Glycine max* (Gm), *Solanum lycopersicum* (Sl), *Arabidopsis lyrata* (Al), *Persea americana* (Pa), *Carica papaya* (Cp), *Cucumis melo* (Cm), *Capsicum annuum* (Ca), *Brachypodium distachyon* (Bd) and *Oryza sativa* (Os). The sequence alignment was carried out using the ClustalW module present in the DNASTAR Lasergene software package (Version 10) with default settings (Gonnet protein weight matrix). Amino acid positions corresponding to SNPs associated with texture parameters are marked with red rectangles while the amino acid changed in H2 and H3 are highlighted in yellow. Residues that match the consensus sequence exactly are indicated as “.”, while insertions introduced to optimize the alignment are indicated as “-“. Click here for file

Additional file 8 **Md-PG1**_**SSR**_**10kd allelism.** The microsatellite allelic profile in two apple varieties, representing the three alleles detected within the assembled apple collection and here named Md-PG1_SSR_10kd_”1” 289 bp), Md-PG1_SSR_10kd-”2” (292 bp) and Md-PG1_SSR_10kd-”3” (298 bp). Click here for file

## References

[B1] Bourne MC Food texture and viscosity: concept and measurement 2002 2 San Diego: Academic Press

[B2] Harker FR Gunson FA Jaeger SR The case for fruit quality: an interpretive review of consumer attitudes, and preferences for apples Postharvest Biology and Technology 2003 28 333 347 10.1016/S0925-5214(02)00215-6

[B3] Vicente AR Saladie M Rose JKC Labavitch JM The linkage between cell wall metabolism and fruit softening: looking to the future J Sci Food Agric 2007 87 8 1435 1448 10.1002/jsfa.2837

[B4] Duizer L A review of acoustic research for studying the sensory perception of crisp, crunchy and crackly textures Trands in Food Science Technology 2001 12 17 24 10.1016/S0924-2244(01)00050-4

[B5] Kilcast D Measuring consumer perceptions of texture: an overview. Texture in food: volume 2: solid foods 2004 3 32

[B6] Giovannoni JJ Molecular biology of fruit maturation and ripening Annual Review of Plant Physiology and Plant Molecular Biology 2001 52 725 749 10.1146/annurev.arplant.52.1.725 11337414

[B7] Costa F Cappellin L Fontanari M Longhi S Guerra W Magnago P Gasperi F Biasioli F Texture dynamics during postharvest storage ripening in apple (Malus x domestica Borkh.) Postharvest Biology and Technology 2012 69 54 63

[B8] Bartley GE Ishida BK Developmental gene regulation during tomato fruit ripening and in-vitro sepal morphogenesis BMC Plant Biol 2003 3 4 10.1186/1471-2229-3-4 12906715 PMC194401

[B9] Costa F Alba R Schouten H Soglio V Gianfranceschi L Serra S Musacchi S Sansavini S Costa G Fei ZJ Giovannoni J Use of homologous and heterologous gene expression profiling tools to characterize transcription dynamics during apple fruit maturation and ripening BMC Plant Biol 2010 10 229 10.1186/1471-2229-10-229 20973957 PMC3095317

[B10] Brummell DA Cell wall disassembly in ripening fruit Funct Plant Biol 2006 33 103 119 10.1071/FP05234 32689218

[B11] Cosgrove DJ Loosening of plant cell walls by expansins Nature 2000 407 321 326 10.1038/35030000 11014181

[B12] Wu Q Szakacs-Dobozi M Hemmat M Hrazdina G Endopolygalacturonase in apple (Malus domestica) and its expression during fruit ripening Plant Physiol 1993 102 219 225 12231813 10.1104/pp.102.1.219 PMC158766

[B13] Hadfield KA Bennett AB Polygalacturonases: amny genes in search of a function Plant Physiol 1998 117 337 343 10.1104/pp.117.2.337 9625687 PMC1539180

[B14] Williatts WGT McCartney L Mackie W Knox PJ Pectin: cell biology and prospects for functional analysis Plant Mol Biol 2001 47 9 27 10.1023/A:1010662911148 11554482

[B15] Mauricio R Mapping quantitative trait loci in plants: uses and caveats for evolutionary biology Nat Rev Genet 2001 2 5 370 381 10.1038/35072085 11331903

[B16] Doerge RW Mapping and analysis of quantitative trait loci in experimental populations Nat Rev Genet 2002 3 1 43 52 11823790 10.1038/nrg703

[B17] Falconer DS Mackay TFC Introduction to quantitative genetics Genetics 2004 167 4 1529 1536 15342495 10.1093/genetics/167.4.1529 PMC1471025

[B18] Mackay TFC Stone EA Ayroles JF The genetics of quantitative traits: challenges and prospects Nat Rev Genet 2009 10 8 565 577 10.1038/nrg2612 19584810

[B19] Holland JB Genetic architecture of complex traits in plants Curr Opin Plant Biol 2007 10 2 156 161 10.1016/j.pbi.2007.01.003 17291822

[B20] Giovannoni JJ Fruit ripening mutants yield insights into ripening control Curr Opin Plant Biol 2007 10 283 289 10.1016/j.pbi.2007.04.008 17442612

[B21] Waldron KW Parker ML Smith AC Plant cell walls and food quality Comprehensive Reviews in Food Science and Food Safety2 2003 2 4 101 119 10.1111/j.1541-4337.2003.tb00019.x 33451229

[B22] King GJ Lynn JR Dover CJ Evans KM Seymour GB Resolution of quantitative trait loci for mechanical measures accounting for genetic variation in fruit texture of apple (Malus pumila Mill) Theor Appl Genet 2001 102 8 1227 1235 10.1007/s001220000530

[B23] Maliepaard C Sillanpaa MJ van Ooijen JW Jansen RC Arjas E Bayesian versus frequentist analysis of multiple quantitative trait loci with an application to an outbred apple cross Theor Appl Genet 2001 103 8 1243 1253 10.1007/s001220100720

[B24] Costa F Stella S Van de Weg WE Guerra W Cecchinel M Dalla Via J Koller B Sansavini S Role of the genes Md-ACO1 and Md-ACS1 in ethylene production and shelf life of apple (Malus domestica Borkh) Euphytica 2005 141 181 190 10.1007/s10681-005-6805-4

[B25] Costa F Van de Weg WE Stella S Dondini L Pratesi D Musacchi S Sansavini S Map position and functional allelic diversity of Md-Exp7, a new putative expansin gene associated with fruit softening in apple (Malus x domestica Borkh.) and pear (Pyrus communis) Tree Genetics & Genomes 2008 4 3 575 586 10.1007/s11295-008-0133-5

[B26] Costa F Peace CP Stella S Serra S Musacchi S Bazzani M Sansavini S Van de Weg WE QTL dynamics for fruit firmness and softening around an ethylene-dependent polygalacturonase gene in apple (Malusxdomestica Borkh) J Exp Bot 2010 61 11 3029 3039 10.1093/jxb/erq130 20462945 PMC2892147

[B27] Longhi S Moretto M Viola R Velasco R Costa F Comprehensive QTL mapping survey dissects the complex fruit texture physiology in apple (Malus x domestica Borkh) J Exp Bot 2012 63 3 1107 1121 10.1093/jxb/err326 22121200

[B28] Flint-Garcia SA Thornsberry JM Buckler ES Structure of linkage disequilibrium in plants Annu Rev Plant Biol 2003 54 357 374 10.1146/annurev.arplant.54.031902.134907 14502995

[B29] Darvasi A Shifman S The beauty of admixture Nat Genet 2005 37 2 118 119 10.1038/ng0205-118 15678141

[B30] Gonzalez-Martinez SC Wheeler NC Ersoz E Nelson CD Neale DB Association genetics in Pinus taeda L. I. Wood property traits Genetics 2007 175 1 399 409 17110498 10.1534/genetics.106.061127 PMC1775017

[B31] Ehrenreich IM Hanzawa Y Chou L Roe JL Kover PX Purugganan MD Candidate gene association mapping of arabidopsis flowering time Genetics 2009 183 1 325 335 10.1534/genetics.109.105189 19581446 PMC2746157

[B32] Waugh R Jannink JL Muehlbauer GJ Ramsay L The emergence of whole genome association scans in barley Curr Opin Plant Biol 2009 12 218 222 10.1016/j.pbi.2008.12.007 19185530

[B33] Ingvarsson PK Street NR Association genetics of complex traits in plants New Phytol 2011 189 4 909 922 10.1111/j.1469-8137.2010.03593.x 21182529

[B34] Myles S Peiffer J Brown PJ Ersoz ES Zhang ZW Costich DE Buckler ES Association mapping: critical considerations shift from genotyping to experimental design Plant Cell 2009 21 8 2194 2202 10.1105/tpc.109.068437 19654263 PMC2751942

[B35] Rafalski A Applications of single nucleotide polymorphisms in crop genetics Curr Opin Plant Biol 2002 5 2 94 100 10.1016/S1369-5266(02)00240-6 11856602

[B36] Mackay I Powell W Methods for linkage disequilibrium mapping in crops Trends Plant Sci 2007 12 2 57 63 10.1016/j.tplants.2006.12.001 17224302

[B37] Rafalski A Association genetics in crop improvement Curr Opin Plant Biol 2010 13 2 174 180 10.1016/j.pbi.2009.12.004 20089441

[B38] Van Ooijen JW JoinMap® 4, Software for the Calculation of Genetic Linkage Maps in Experimental Populations 2006 Netherlands: Kyazma B.V, Wageningen

[B39] Van Ooijen JW MAPQTL® 6, Software for the mapping of quantitative trait loci in experimental populations of diploid species 2009 Netherlands: Kyazma B.V, Wageningen

[B40] Vorrips RE MapChart: software for the graphical presentation of linkage maps and QTLs J Hered 2002 93 77 78 10.1093/jhered/93.1.77 12011185

[B41] Price AL Principal component analysis corrects for stratification in genome-wide association stidies Nat Genet 2006 38 904 909 10.1038/ng1847 16862161

[B42] Zhang Z Buckler ES Casstevens TM Bradbury PJ Software engineering the mixed model for genome-wide association studies on large samples Brief Bioinform 2009 10 6 664 675 10.1093/bib/bbp050 19933212

[B43] Yu JM Pressoir G Briggs WH A unified mixed-model method for association mapping that accounts for multiple levels of relatedness Nat Genet 2006 38 2 203 208 10.1038/ng1702 16380716

[B44] Bradbury PJ Zhang Z Kroon DE Casstevens TM Ramdoss Y Buckler ES TASSEL: software for association mapping of complex traits in diverse samples Bioinformatics 2007 23 19 2633 2635 10.1093/bioinformatics/btm308 17586829

[B45] Barrett JC Fry B Maller J Daly MJ Haploview: analysis and visualization of LD and haplotype maps Bioinformatics 2005 21 2 263 265 10.1093/bioinformatics/bth457 15297300

[B46] Purcell S Neale B Todd-Brown K PLINK: a tool set for whole-genome association and population-based linkage analyses Am J Hum Genet 2007 81 3 559 575 10.1086/519795 17701901 PMC1950838

[B47] Storey JD Tibshirani R Statistical significance for genomewide studies PNAS 2003 100 16 9440 9445 10.1073/pnas.1530509100 12883005 PMC170937

[B48] Scheet P Stephens M A fast and flexible statistical model for large-scale population genotype data: applications to inferring missing genotypes and haplotypic phase Am J Hum Genet 2006 78 4 629 644 10.1086/502802 16532393 PMC1424677

[B49] Velasco R Zharkikh A Affourtit J The genome of the domesticated apple (Malus x domestica Borkh) Nat Genet 2010 42 10 833 839 10.1038/ng.654 20802477

[B50] Chagnè D Gasic K Crowhurst RN Han Y Bassett HC Bowatte DR Lawrence TJ Rikkerink EHA Gardiner SE Korban SS Development of a set of SNP markers present in expressed genes of the apple Genomics 2008 92 5 353 358 10.1016/j.ygeno.2008.07.008 18721872

[B51] Dantec LL Chagne D Pot D Automated SNP detection in expressed sequence tags: statistical considerations and application to maritime pine sequences Plant Mol Biol 2004 54 3 461 470 15284499 10.1023/B:PLAN.0000036376.11710.6f

[B52] Cogan NOI Ponting RC Vecchies AC Gene-associated single nucleotide polymorphism discovery in perennial ryegrass (Lolium perenne L.) Mol Genet Genomics 2006 276 2 101 112 10.1007/s00438-006-0126-8 16708235

[B53] Lijavetzky D Cabezas JA Ibanez A Rodriguez V Martinez-Zapater JM High throughput SNP discovery and genotyping in grapevine (Vitis vinifera L.) by combining a re-sequencing approach and SNPlex technology BMC Genomic 2007 8 424 10.1186/1471-2164-8-424 PMC2212664 18021442

[B54] Kolkman JM Berry ST Leon AJ Slabaugh MB Tang S Gao W Shintani DK Burke JM Knapp SJ Single nucleotide polymorphisms and linkage disequilibrium in sunflower Genetics 2007 177 1 457 468 10.1534/genetics.107.074054 17660563 PMC2013689

[B55] Bird CR Smith CJS Ray JA Moureau P Bevan MW Bird AS Hughes S Morris PC Grierson D Schuch W The tomato polygalacturonase gene and ripening-specific expression in transgenic plants Plant Mol Biol 1988 11 5 651 662 10.1007/BF00017465 24272499

[B56] Atkinson RG Gardner RC A polygalacturonase gene from kiwifruit (Actinidia-Deliciosa) Plant Physiol 1993 103 2 669 670 10.1104/pp.103.2.669 8029342 PMC159031

[B57] Breseghello F Sorells ME Association mapping of kernel size and milling quality in wheat (Triticum aestivum L.) cultivars Genetics 2006 172 1165 1177 16079235 10.1534/genetics.105.044586 PMC1456215

[B58] Gupta PK Rustgi S Kulwal PL Linkage disequilibrium and association studies in higher plants: present status and future prospects Plant Mol Biol 2005 57 4 461 485 10.1007/s11103-005-0257-z 15821975

