# Supplementary material for: A candidate gene based approach validates Md-PG1 as the main responsible for a QTL impacting fruit texture in apple (Malus x domestica Borkh)
Source: BMC Plant Biol. 2013 Mar 4;13:37. doi: 10.1186/1471-2229-13-37 (PMC3599472; doi:10.1186/1471-2229-13-37)
Supplement: Additional file 1 — List of primers used for gene cloning and sequencing as well as sequences of the two microsatellite markers identified within theMd-PG1region, and located at 3 kb upstream (Md-PG1SSR) and 10 kb downstream (Md-PG1SSR10kd) from the start and stop codon, respectively. [file 1471-2229-13-37-S1.doc]

| **Primer** | **Forward** | **Reverse** |
| --- | --- | --- |
| PG_full | ACCTCAAGAGCCCAAGACGACACAAT | CTCGGGACTTCTTCCAACAATGTAGAAATGGTCG |
| PG_1 | AGGTCAACGCGCTTCATAGT | ACCACAAGAACCATAGCTCCA |
| PG_2 | GAAGGCAGCTTGTTCTTCCA | TGCACCTGTTGAAGGTCACA |
| PG_3 | ATGTGCAGGCTGTGACCTTC | CCGTCTTCTCCCAAGCTACC |
| PG_4 | AGAAAAGTGCGGGATGACTTT | TCTAGGGGAGACAACTCCTTTG |
| PG_1ku | GTTGACAATCCAAAAATATCATCC | TGATGACAAAGAGAACGAACG |
| PG_1ku2 | GACATAGACCACTGGCTCATTA | AGACTAGGGTTGGAAATTACC |
| PG_1kb_up_Gold | TATGGTTCTTGTGGTACCAAG | AGACTAGGGTTGGAAATTACC |
| PG_1kd | CGACTAGCCTAACCCCAGC | CTTCTGCTGCTGTGTTGTGC |
| Md-PG1SSR | CTTGCTTAAACCGCATGCTT | AAATTGAGGCACGTGATGGT |
| Md-PG1SSR_10kd | TTTCTTCCTTGGGTTTTTGG | ACTCGTGCGCCAGATAGC |
