# Supplementary material for: A candidate gene based approach validates Md-PG1 as the main responsible for a QTL impacting fruit texture in apple (Malus x domestica Borkh)
Source: BMC Plant Biol. 2013 Mar 4;13:37. doi: 10.1186/1471-2229-13-37 (PMC3599472; doi:10.1186/1471-2229-13-37)
Supplement: Additional file 2 — List of 17 SSRs used to compute the population structure. For each marker the forward and reverse primer sequences and linkage group are provided. For the list of SNPs see Longhi et al. [27]. [file 1471-2229-13-37-S2.doc]

| **Name** | **Forward** | **Reverse** | **LG** |
| --- | --- | --- | --- |
| CH05G08 | CCAAGACCAAGGCAACATTT | CCCTTCACCTCATTCTCACC | 1 |
| CH05E03 | CGAATATTTTCACTCTGACTGGG | CAAGTTGTTGTACTGCTCCGAC | 2 |
| CH03G07 | AATAAGCATTCAAAGCAATCCG | TTTTTCCAAATCGAGTTTCGTT | 3 |
| Hi23G02 | TTTTCCAGGATATACTACCCTTCC | GTTTCTTCGAGGTCAGGGTTTG | 4 |
| CH04E03 | TTGAAGATGTTTGGCTGTGC | TGCATGTCTGTCTCCTCCAT | 5 |
| CH03D12 | GCCCAGAAGCAATAAGTAAACC | ATTGCTCCATGCATAAAGGG | 6 |
| Hi03A10 | GGACCTGCTTCCCCTTATTC | CAGGGAACTTGTTTGATGG | 7 |
| CH01C06 | TTCCCCATCATCGATCTCTC | AAACTGAAGCCATGAGGGC | 8 |
| CH01F03b | GAGAAGCAAATGCAAAACCC | CTCCCCGGCTCCTATTCTAC | 9 |
| CH02b03b | ATAAGGATACAAAAACCCTACACAG | GACATGTTTGGTTGAAAACTTG | 10 |
| CH02D08 | TCCAAAATGGCGTACCTCTC | GCAGACACTCACTCACTATCTCTC | 11 |
| CH01G12 | CCCACCAATCAAAAATCACC | TGAAGTATGGTGGTGCGTTC | 12 |
| CH05H05 | ACATGTCACTCCTACGCGG | GTGCAGTGATTAGCATTGCTGT | 13 |
| CH01G05 | CATCAGTCTCTTGCACTGGAAA | GACAGAGTAAGCTAGGGCTAGGG | 14 |
| NZ02B1 | CCGTGATGACAAAGTGCATGA | ATGAGTTTGATGCCCTTGGA | 15 |
| CH04F10 | GTAATGGAAATACAGTTTCACAA | TTAAATGCTTGGTGTGTTTTGC | 16 |
| GD96 | CGGCGGAAAGCAATCACCT | GCCAGCCCTCTATGGTTCCAGA | 17 |
