# Supplementary figures and images for: A candidate gene based approach validates Md-PG1 as the main responsible for a QTL impacting fruit texture in apple (Malus x domestica Borkh)
Source: BMC Plant Biol. 2013 Mar 4;13:37. doi: 10.1186/1471-2229-13-37 (PMC3599472; doi:10.1186/1471-2229-13-37)

## Slide 1
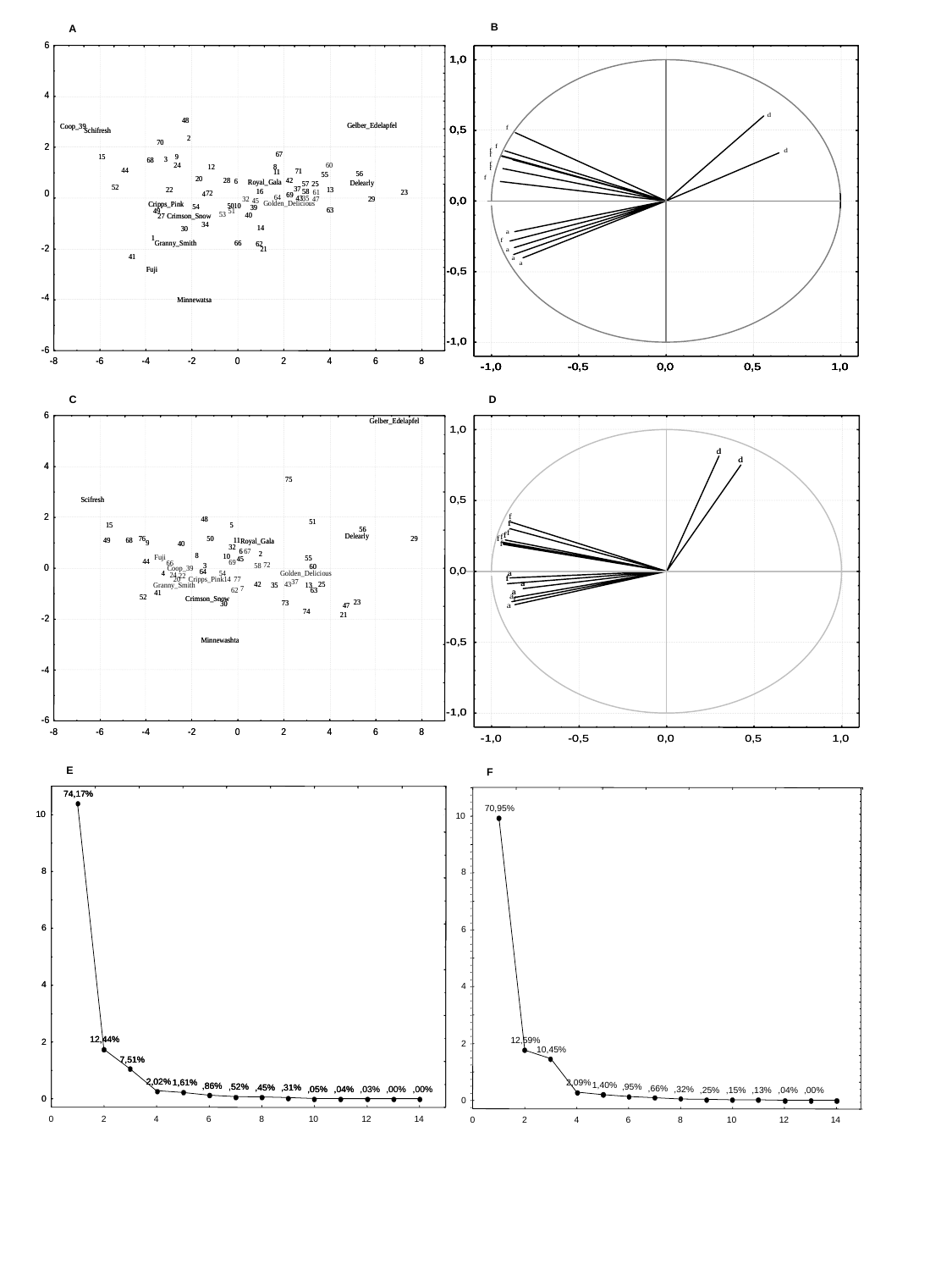

B
A
C
D
E
F
74,17%
74,17%
74,17%
10
10
8
8
6
6
4
4
12,44%
12,44%
12,44%
2
2
7,51%
7,51%
7,51%
2,02%
2,02%
2,02%
1,61%
1,61%
1,61%
,86%
,86%
,86%
,52%
,52%
,52%
,45%
,45%
,45%
,31%
,31%
,31%
,05%
,05%
,05%
,04%
,04%
,04%
,03%
,03%
,00%
,00%
,00%
,00%
0
0
0
2
4
6
8
10
12
14
70,95%
10
8
6
4
12,59%
2
10,45%
2,09%
1,40%
,95%
,66%
,32%
,25%
,15%
,13%
,04%
,00%
0
0
2
4
6
8
10
12
14

Supplement: Additional file 3 — PCA plot (first two dimensions) distribution of the varieties assessed with the novel texture phenomics strategy at postharvest stage for year 1 (A) and 2 (C) respectively. On the variables projection (panel B and D) “a” and “f” means acoustic and force parameters respectively, while “d” is for force direction. Panels E and F show the eigenvalue for the two years of assessment. Numbers: variety codes as reported in Table 1. [file 1471-2229-13-37-S3.ppt]

## Slide 1
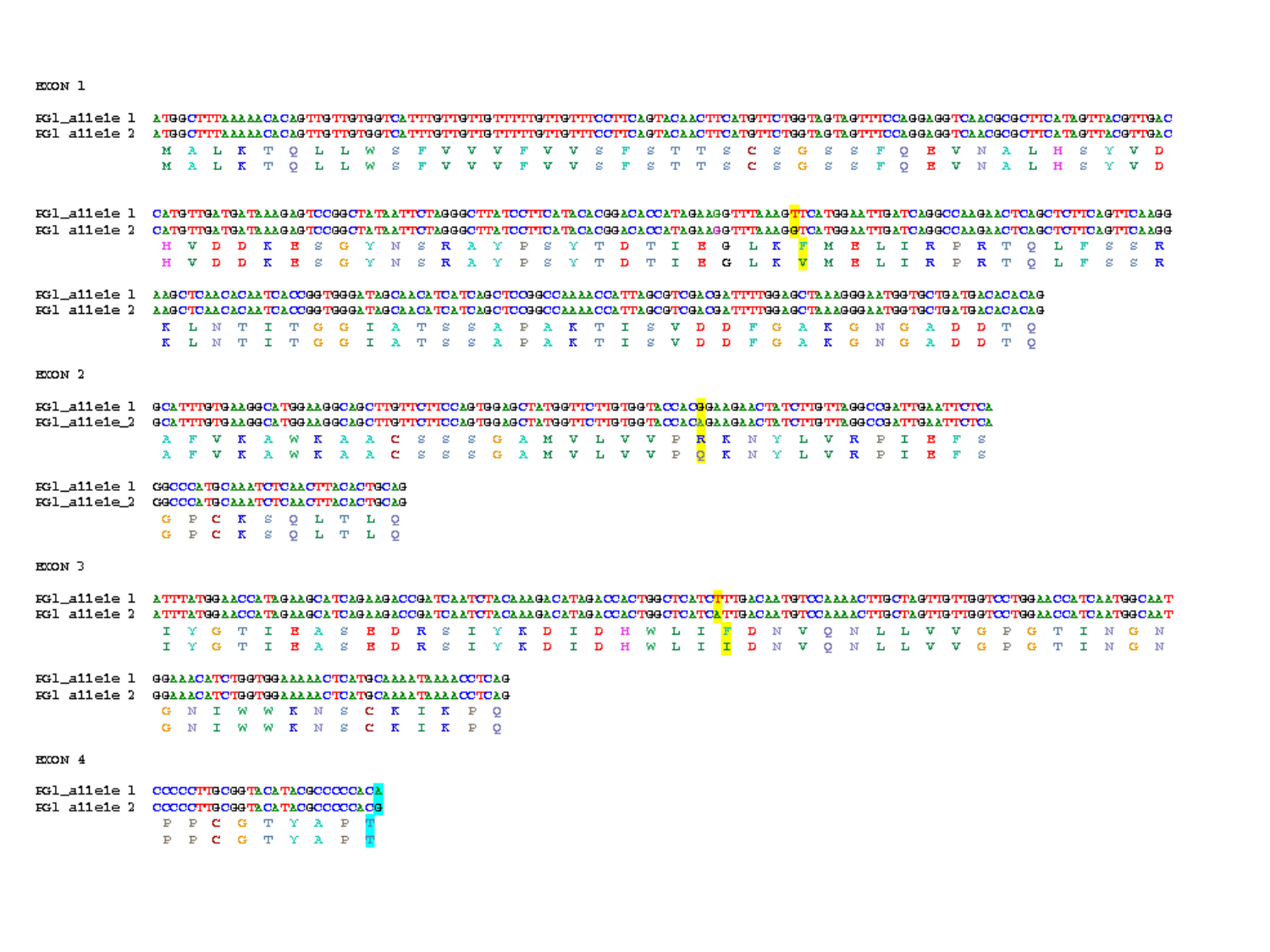

## Slide 2
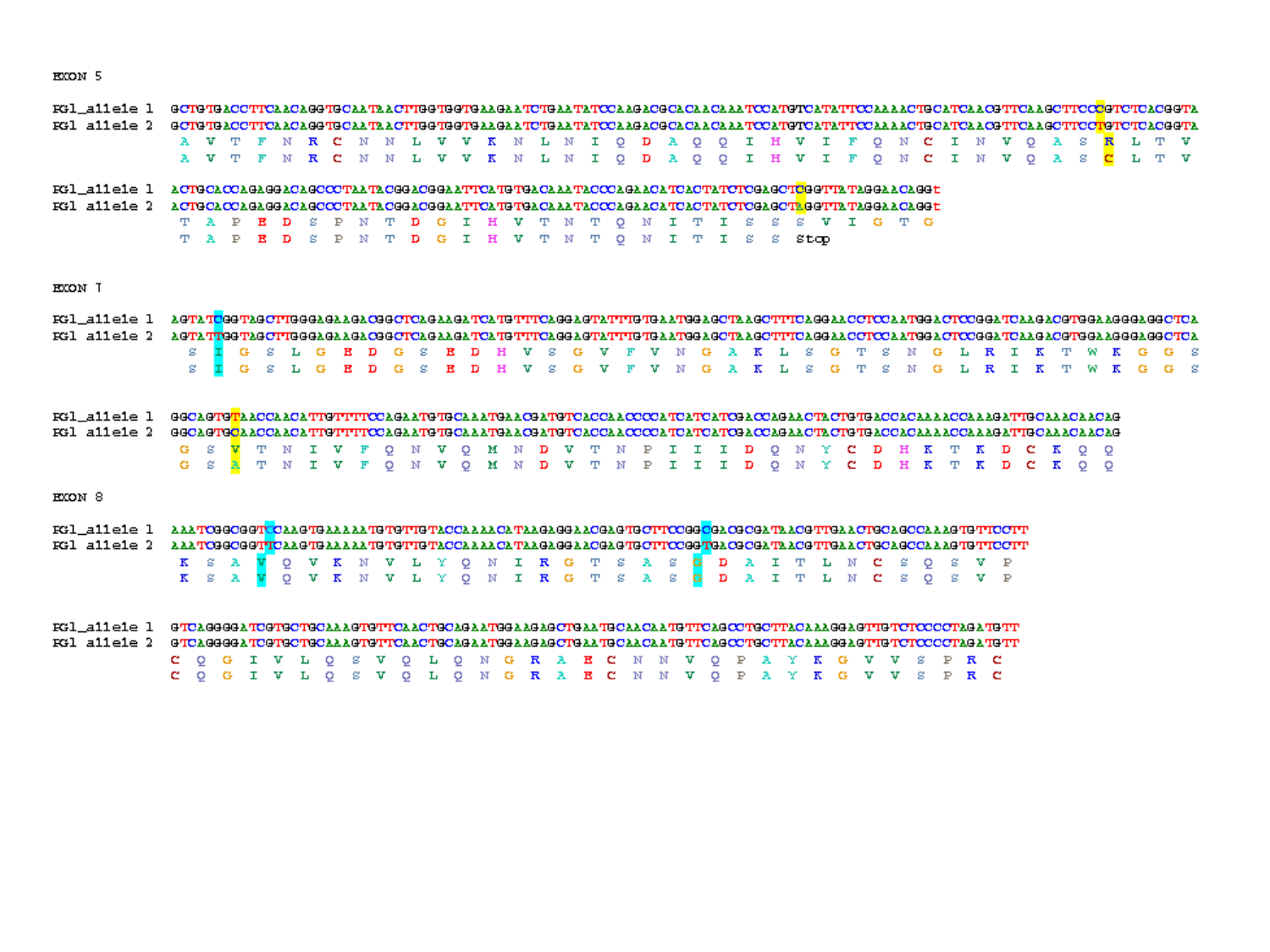

Supplement: Additional file 5 — Md-PG1 amino acid sequence derived by the predicted Md-PG1 sequence. The synonymous substitutions are shown in blue, while the non-synonymous substitutions are shown in yellow. (PPT 179 kb) [file 1471-2229-13-37-S5.ppt]

## Slide 1
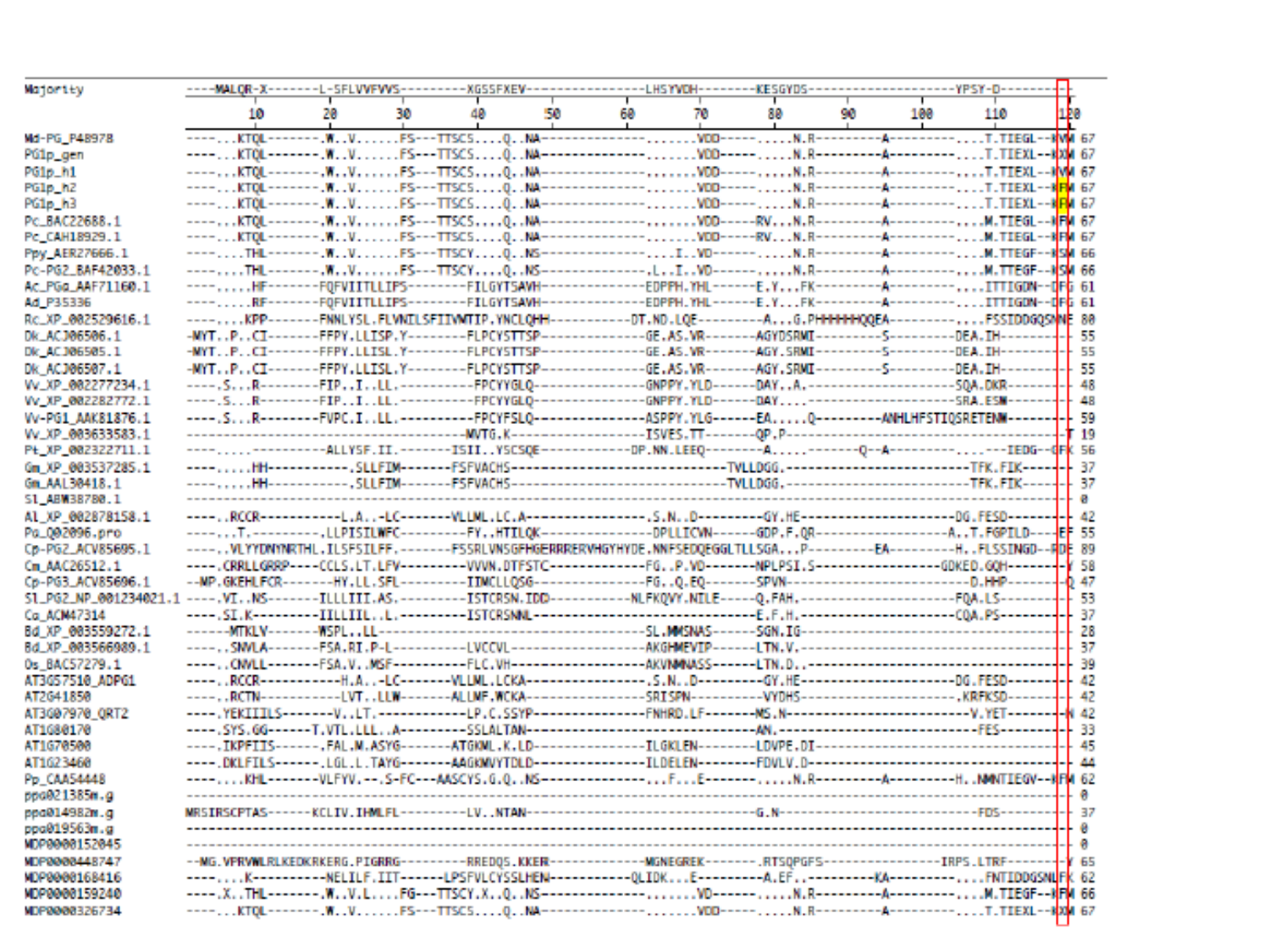

## Slide 2
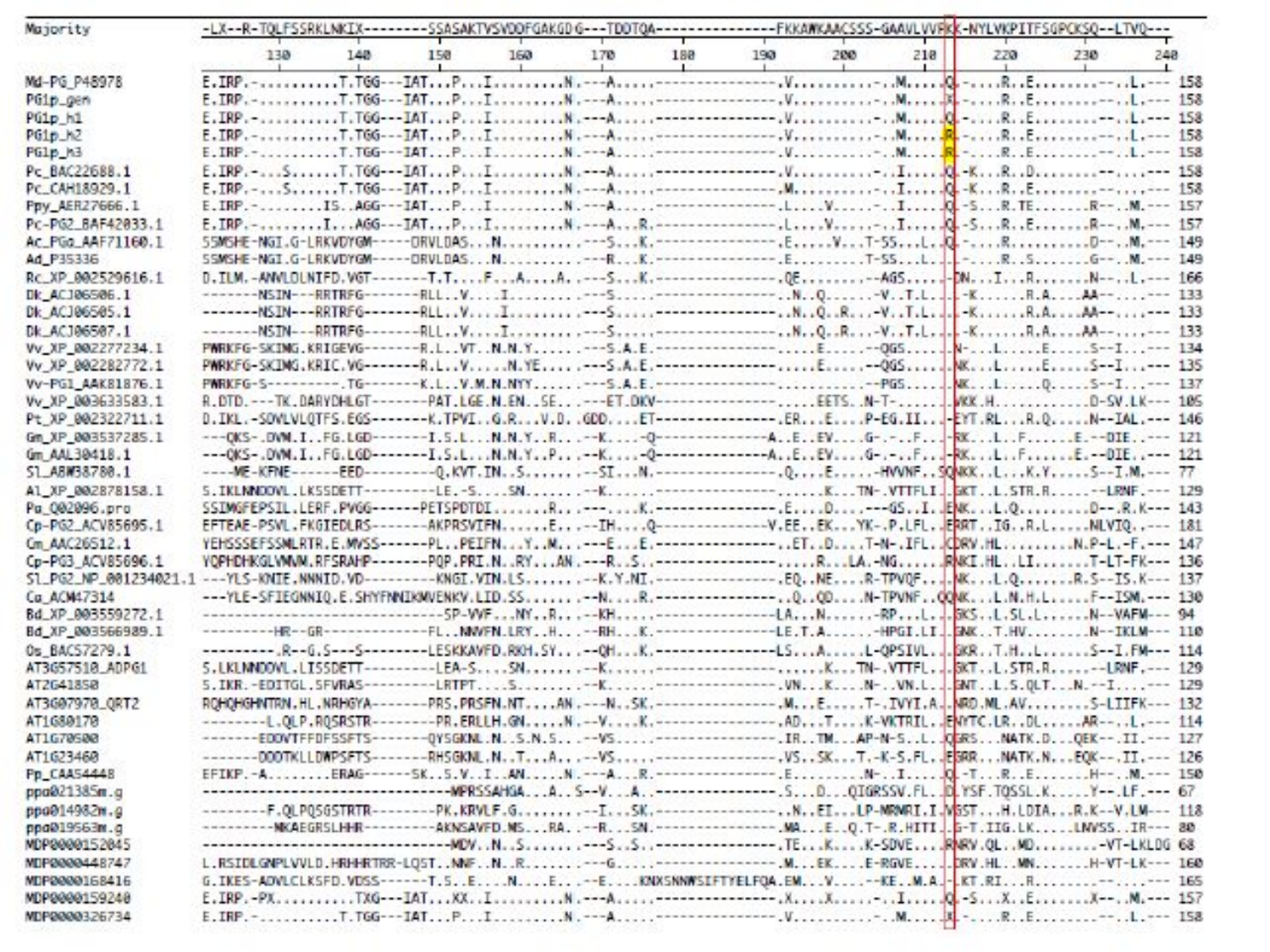

## Slide 3
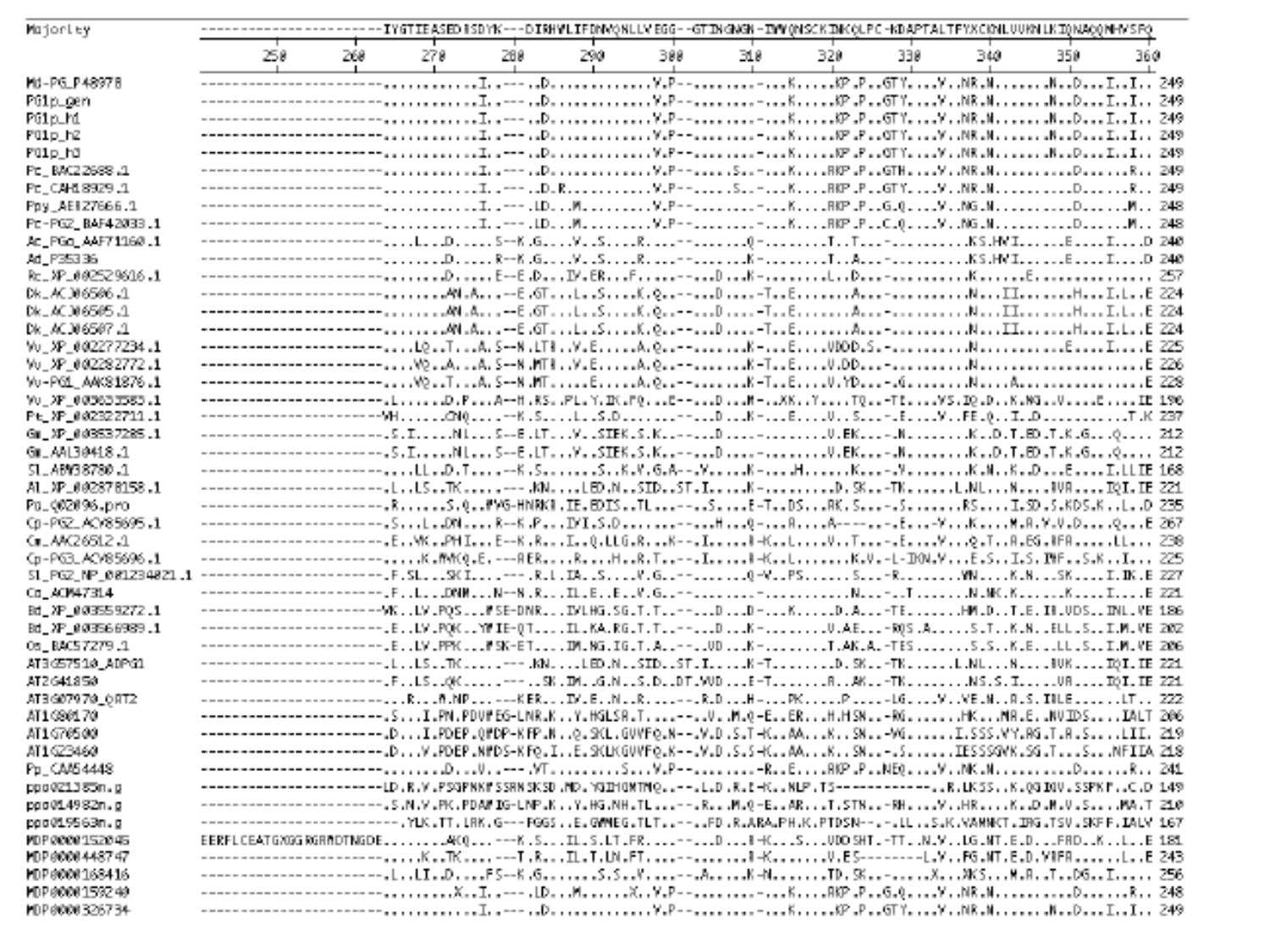

## Slide 4
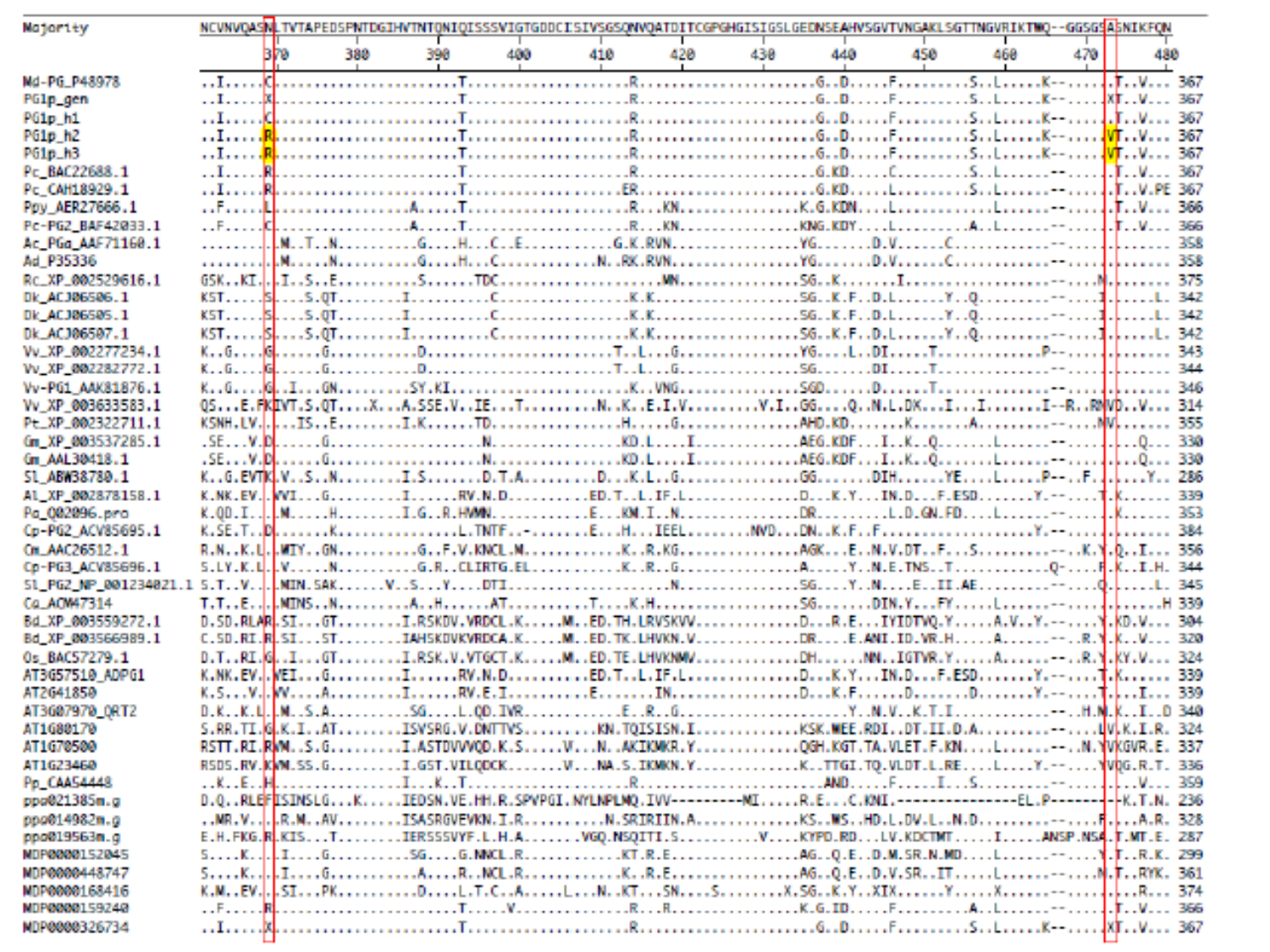

## Slide 5
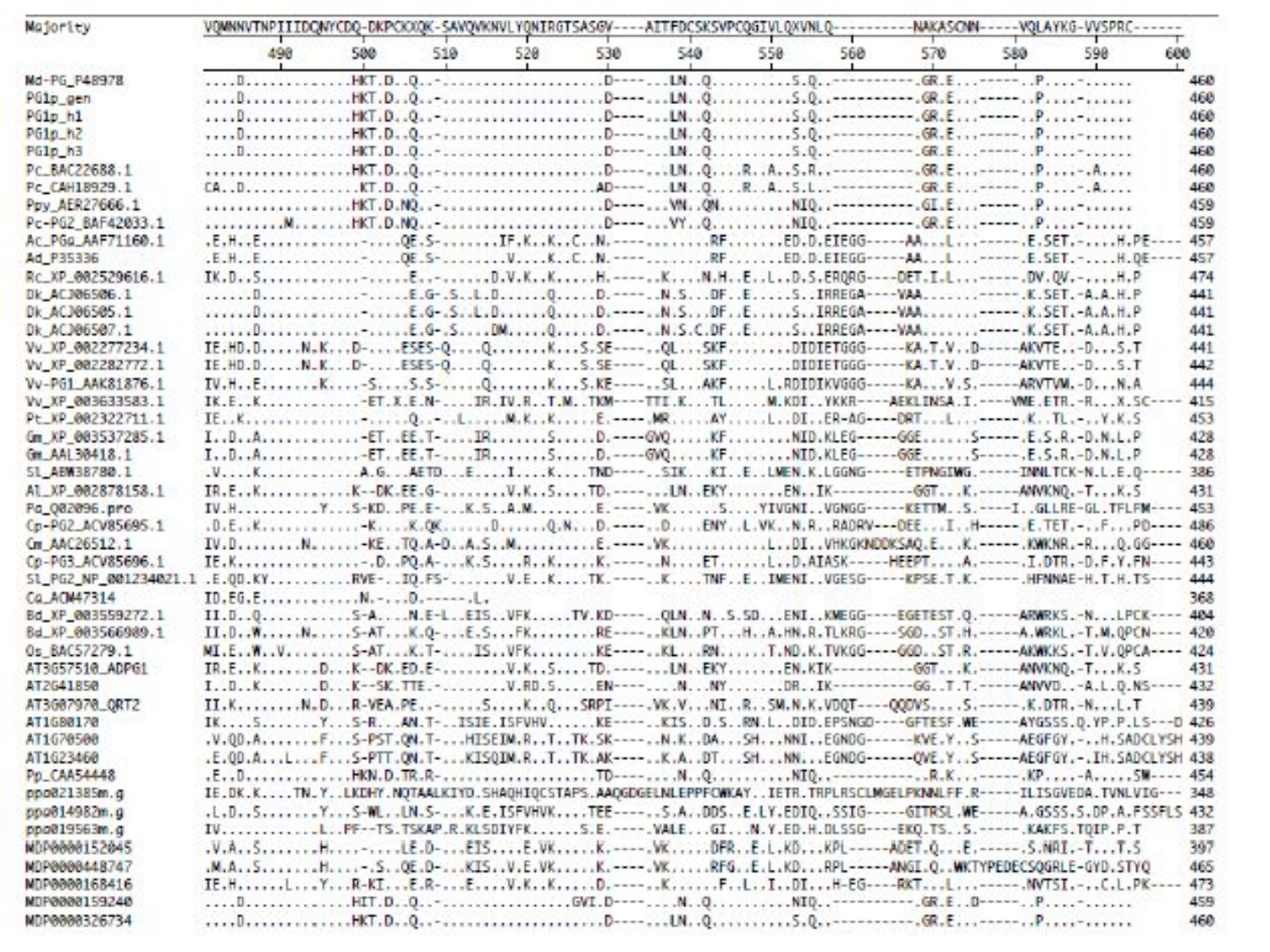

## Slide 6
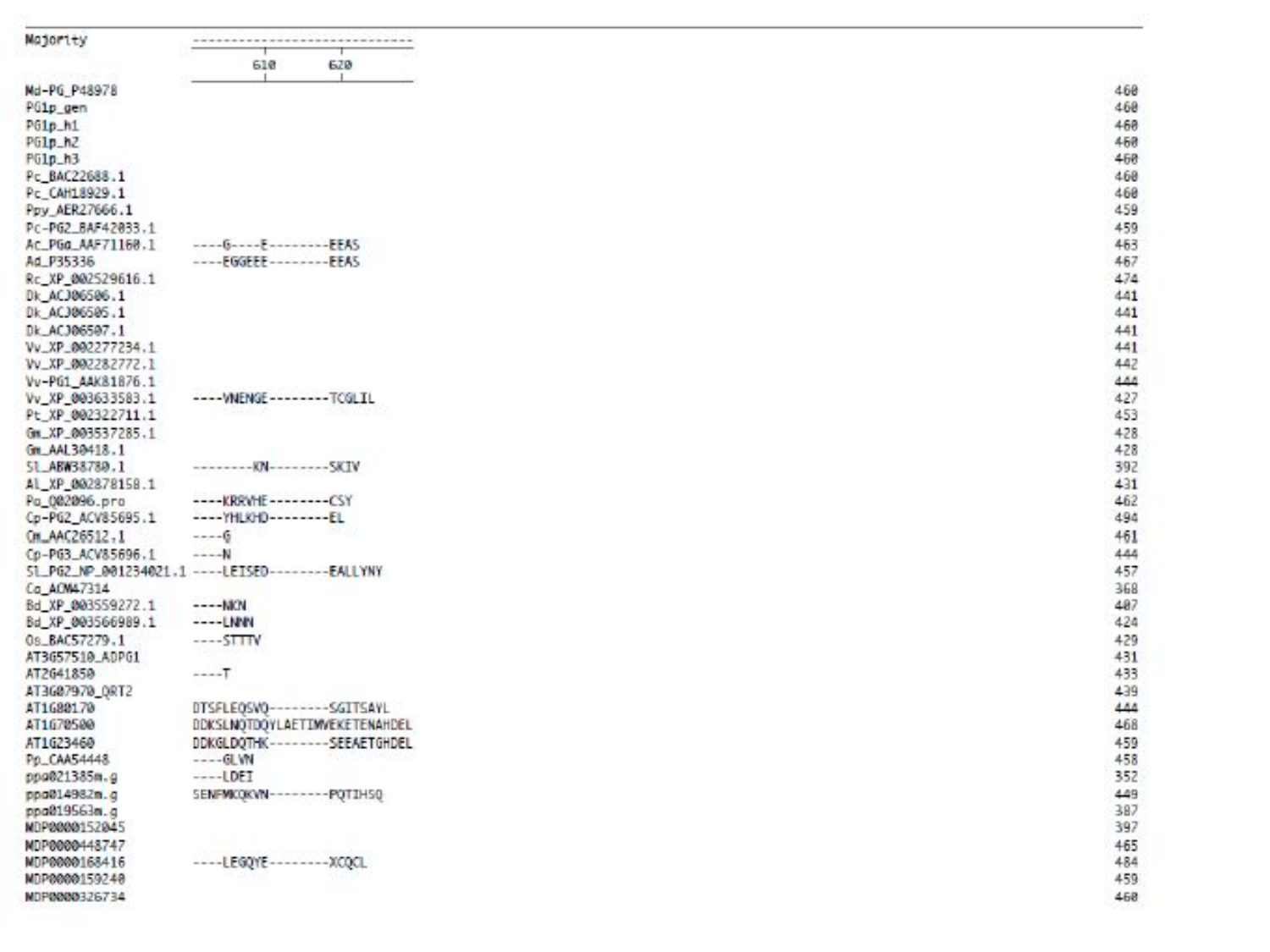

Supplement: Additional file 7 — Amino acid sequence alignment of the different PG enzymes derived from Md-PG1 (MDP0000326734) alleles with other plant PGs. The consensus Md-PG1 sequence is here indicated as PG1p-gen and is aligned with the amino acid sequence of the three haplotypes H1, H2 and H3 (here indicated as PG1p_h1, PG1p_h2 and PG1p_h3, respectively) and the Md-PG1 sequence already present in public databases (Acc. no.: P48978). Other sequences are indicated with the species initials followed by the Acc. nos (e.g.: Pc_BAC22688.1 stands for Pyrus communis BAC22688.1). Apple, (Malus xdomestica, MDP), peach (Prunus persica, ppa) and Arabidopsis (Arabidopsis thaliana, AT) sequences have been downloaded from Phytozome (http://www.phytozome.net), while the others have been downloaded from NCBI (http://www.ncbi.nlm.nih.gov/entrez). Species used in the alignment are: Malus xdomestica (Md), Pyrus communis (Pc), P. pyrifolia (Ppy), Actinidia chinensis (Ac), A. deliciosa (Ad), Prunus persica (Pp), Ricinus communis (Rc), Diospyros kaki (Dk), Vitis vinifera (Vv), Populus trichocarpa (Pt), Glycine max (Gm), Solanum lycopersicum (Sl), Arabidopsis lyrata (Al), Persea americana (Pa), Carica papaya (Cp), Cucumis melo (Cm), Capsicum annuum (Ca), Brachypodium distachyon (Bd) and Oryza sativa (Os). The sequence alignment was carried out using the ClustalW module present in the DNASTAR Lasergene software package (Version 10) with default settings (Gonnet protein weight matrix). Amino acid positions corresponding to SNPs associated with texture parameters are marked with red rectangles while the amino acid changed in H2 and H3 are highlighted in yellow. Residues that match the consensus sequence exactly are indicated as “.”, while insertions introduced to optimize the alignment are indicated as “-“. [file 1471-2229-13-37-S7.ppt]

## Slide 1
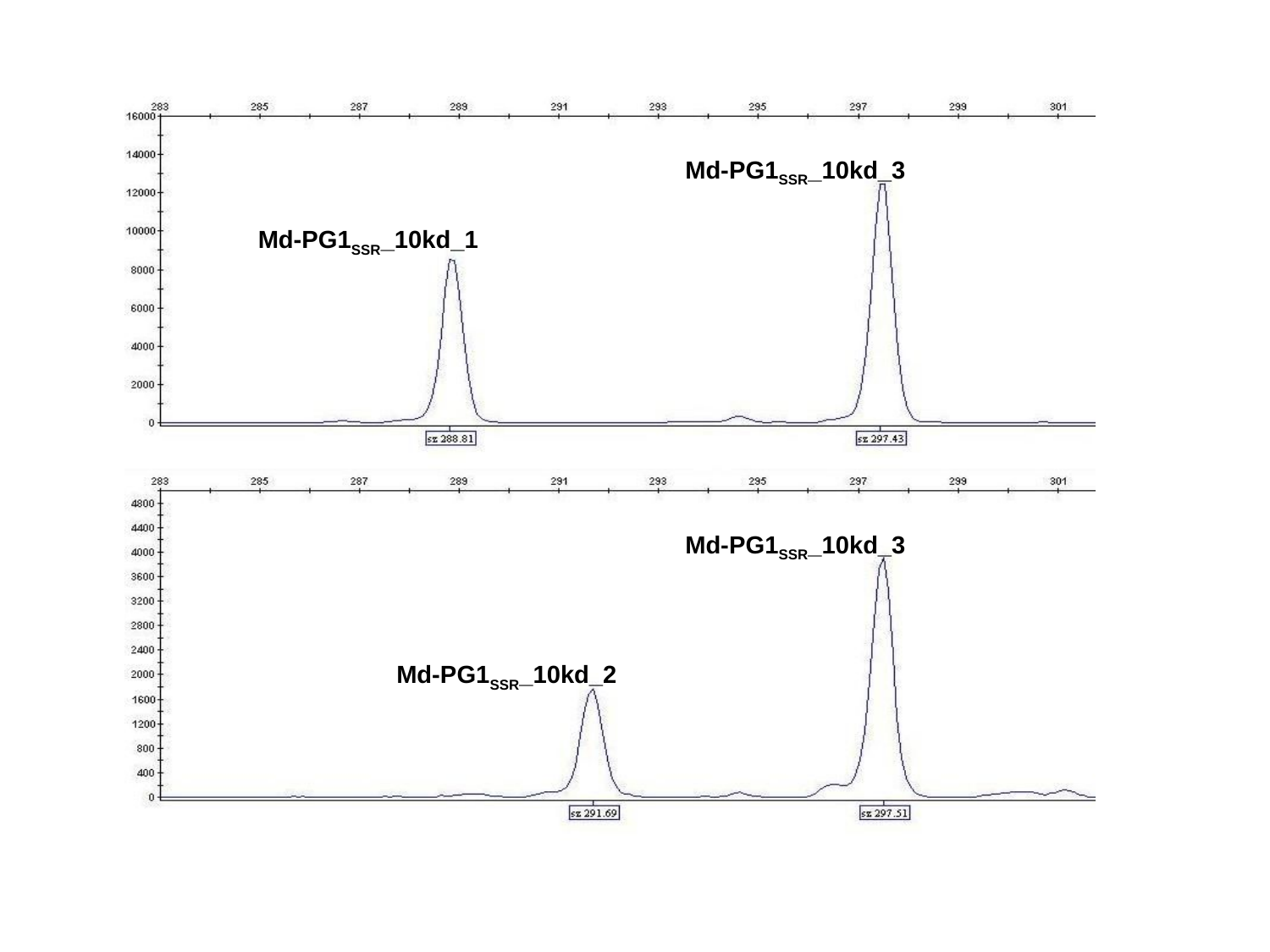

Md-PG1SSR_10kd_3
Md-PG1SSR_10kd_1
Md-PG1SSR_10kd_3
Md-PG1SSR_10kd_2

Supplement: Additional file 8 — Md-PG1SSR10kd allelism. The microsatellite allelic profile in two apple varieties, representing the three alleles detected within the assembled apple collection and here named Md-PG1SSR10kd_”1” 289 bp), Md-PG1SSR10kd-”2” (292 bp) and Md-PG1SSR10kd-”3” (298 bp). [file 1471-2229-13-37-S8.ppt]
