# Supplementary material for: A candidate gene based approach validates Md-PG1 as the main responsible for a QTL impacting fruit texture in apple (Malus x domestica Borkh)
Source: BMC Plant Biol. 2013 Mar 4;13:37. doi: 10.1186/1471-2229-13-37 (PMC3599472; doi:10.1186/1471-2229-13-37)
Supplement: Additional file 4 — SNP organization withinMd-PG1gene. For each SNP used in the association analysis the location with respect to the gene structure (location), the type of aminoacid substitution (type; nc: non-coding, ns: non-synonymous, s: synonymous) and the change of aminoacid and physical position (in bp after the start codon ATG) are reported. [file 1471-2229-13-37-S4.doc]

| *SNP* | *location* | *type* | *aa change* | *bp after ATG* |
| --- | --- | --- | --- | --- |
| pg_full_1 | exon_1 | ns | V/F | 196 |
| pg_full_2 | intron | nc | - | 355 |
| pg_full_3 | intron | nc | - | 387 |
| pg_full_4 | intron | nc | - | 466 |
| pg_full_5 | intron | nc | - | 469 |
| pg_full_6 | exon_2 | ns | Q/R | 703 |
| pg_full_7 | intron | nc |  | 824 |
| pg_full_8 | exon_3 | ns | F/I | 944 |
| pg_full_9 | exon_4 | s | T/T | 1146 |
| pg_full_10 | exon_5 | ns | C/R | 1358 |
| pg_full_11 | exon_5 | ns | S/Stop | 1143 |
| pg_full_12 | intron | nc | - | 1478 |
| pg_full_13 | intron | nc | - | 1507 |
| pg_full_14 | intron | nc | - | 1508 |
| pg_full_15 | intron | nc | - | 1776 |
| pg_full_16 | intron | nc | - | 1780 |
| pg_full_17 | exon_7 | s | I/I | 1877 |
| pg_full_18 | exon_7 | ns | A/V | 1999 |
| pg_full_19 | intron | nc | - | 2133 |
| pg_full_20 | intron | nc | - | 2180 |
| pg_full_21 | exon_8 | s | V/V | 2208 |
| pg_full_22 | exon_8 | s | G/G | 2259 |
