# Supplementary material for: A candidate gene based approach validates Md-PG1 as the main responsible for a QTL impacting fruit texture in apple (Malus x domestica Borkh)
Source: BMC Plant Biol. 2013 Mar 4;13:37. doi: 10.1186/1471-2229-13-37 (PMC3599472; doi:10.1186/1471-2229-13-37)
Supplement: Additional file 6 — Significant marker – texture sub-traits association computed with MLM algorithm and corrected by FDR < 0.05. Only significant values (P-value < 0.05) are reported. Ac is for Acoustic parameter, while F is for Force-mechanical parameters. [file 1471-2229-13-37-S6.doc]

| *Marker* | *Ac_Linear_Distance* | *Ac_Peack* | *Max_Ac_Pressure* | *Mean_Ac_Pressure* | *Area* | *Final_F* | *F_Linear_Distance* | *F_Peack* | *Max_F* | *Mean_F* | *Yield_F* | *Young_Module* | *R_F* | *∆_F* |
| --- | --- | --- | --- | --- | --- | --- | --- | --- | --- | --- | --- | --- | --- | --- |
| pg_full_1 | 0,0266 | 0,0266 |  |  | 0,0284 | 0,0266 |  | 0,0447 | 0,0284 | 0,0282 | 0,0405 |  |  | 0,0284 |
| pg_full_6 | 0,0266 | 0,0266 |  |  | 0,0266 | 0,0282 |  | 0,0367 | 0,0267 | 0,0266 | 0,0267 |  |  |  |
| pg_full_9 |  | 0,0484 | 0,0266 | 0,0266 | 0,0483 |  | 0,0266 |  | 0,0462 | 0,0483 | 0,0442 |  |  |  |
| pg_full_10 | 0,0266 | 0,0266 |  |  | 0,0266 | 0,0267 |  | 0,0408 | 0,0266 | 0,0266 | 0,0272 |  |  |  |
| pg_full_12 | 0,0266 | 0,0266 |  |  | 0,0266 | 0,0266 |  | 0,0484 | 0,0266 | 0,0266 | 0,0284 |  |  | 0,0447 |
| pg_full_13 |  |  |  |  |  |  |  |  |  |  |  |  | 0,0266 | 0,0266 |
| pg_full_17 |  | 0,0359 |  |  |  |  |  |  |  |  |  |  |  |  |
| pg_full_18 | 0,0266 | 0,0266 |  |  | 0,0445 | 0,0443 |  | 0,0442 | 0,0483 | 0,0442 | 0,0490 |  |  |  |
| pg_full_19 | 0,0266 | 0,0266 |  |  | 0,0311 | 0,0295 |  | 0,0447 | 0,0327 | 0,0295 | 0,0463 |  |  |  |
| pg_full_20 | 0,0295 | 0,0266 |  |  |  |  |  |  |  |  |  |  |  |  |
| pg_full_21 | 0,0266 | 0,0266 |  |  | 0,0266 | 0,0267 |  | 0,0408 | 0,0266 | 0,0266 | 0,0272 |  |  |  |
| pg_23_UTR | 0,0266 | 0,0266 |  |  | 0,0442 | 0,0442 |  | 0,0442 | 0,0442 | 0,0442 |  |  |  |  |
| 1KB_DOWN_1 | 0,0493 | 0,0408 |  |  |  |  |  |  |  |  |  |  |  |  |
| 1KB_DOWN_4 | 0,0266 | 0,0266 |  |  |  |  |  | 0,0356 |  |  |  |  |  |  |
| 1KB_DOWN_5 | 0,0266 | 0,0266 | 0,0404 | 0,0484 | 0,0470 | 0,0490 | 0,0490 |  | 0,0404 | 0,0462 | 0,0462 |  |  |  |
| 1KB_DOWN_7 | 0,0352 | 0,0282 |  |  | 0,0442 |  |  |  | 0,0442 | 0,0442 | 0,0490 |  |  |  |
| 1KB_DOWN_9 |  |  |  |  |  | 0,0483 |  |  |  |  |  |  |  |  |
| 1KB_DOWN_10 |  |  |  |  |  | 0,0404 |  |  |  |  |  |  |  |  |
| Mg-PG1SSR_10kd-2 | 0,0266 | 0,0266 |  |  | 0,0266 | 0,0284 |  | 0,0267 | 0,0276 | 0,0266 | 0,0284 |  | 0,0359 |  |
| Mg-PG1SSR_10kd-3 | 0,0266 | 0,0266 |  |  | 0,0266 | 0,0266 |  | 0,0295 | 0,0266 | 0,0266 | 0,0266 | 0,0447 | 0,0483 | 0,0282 |
